# Investigation on changes of modularity and robustness by edge-removal mutations in signaling networks

**DOI:** 10.1186/s12918-017-0505-2

**Published:** 2017-12-21

**Authors:** Cong-Doan Truong, Yung-Keun Kwon

**Affiliations:** 1 Department of Electrical/Electronic and Computer Engineering, University of Ulsan, 93 Daehak-ro, Nam-gu, Ulsan, 44610 Republic of Korea; 2 Faculty of Information Technology, Hanoi Open University, Hanoi, Vietnam

**Keywords:** Boolean dynamics, Edge-removal mutations, Robustness, Modularity, Feedback loops, Centrality, Gene-ontology, Drug-targets

## Abstract

**Background:**

Biological networks consisting of molecular components and interactions are represented by a graph model. There have been some studies based on that model to analyze a relationship between structural characteristics and dynamical behaviors in signaling network. However, little attention has been paid to changes of modularity and robustness in mutant networks.

**Results:**

In this paper, we investigated the changes of modularity and robustness by edge-removal mutations in three signaling networks. We first observed that both the modularity and robustness increased on average in the mutant network by the edge-removal mutations. However, the modularity change was negatively correlated with the robustness change. This implies that it is unlikely that both the modularity and the robustness values simultaneously increase by the edge-removal mutations. Another interesting finding is that the modularity change was positively correlated with the degree, the number of feedback loops, and the edge betweenness of the removed edges whereas the robustness change was negatively correlated with them. We note that these results were consistently observed in randomly structure networks. Additionally, we identified two groups of genes which are incident to the highly-modularity-increasing and the highly-robustness-decreasing edges with respect to the edge-removal mutations, respectively, and observed that they are likely to be central by forming a connected component of a considerably large size. The gene-ontology enrichment of each of these gene groups was significantly different from the rest of genes. Finally, we showed that the highly-robustness-decreasing edges can be promising edgetic drug-targets, which validates the usefulness of our analysis.

**Conclusions:**

Taken together, the analysis of changes of robustness and modularity against edge-removal mutations can be useful to unravel novel dynamical characteristics underlying in signaling networks.

**Electronic supplementary material:**

The online version of this article (10.1186/s12918-017-0505-2) contains supplementary material, which is available to authorized users.

## Background

Robustness and modularity are key properties to understand complex dynamics in large-scale biological networks. The former means the capability of a network to maintain functioning against external and internal perturbations [1], and the latter describes the divisibility of a network into clusters [2]. The robust dynamics [3–5] and the modularized structures [6–8] have been ubiquitously observed through various biological examples. It is also notable that these properties can be changed by structural mutations because they are highly dependent on the network structure. For example, a few studies showed that the modularity is greatly changed by the removal of hubs [9] or by stabilizing events in protein–protein interaction networks. Some other studies also proved that the robustness is considerably changeable according to a variety of mutations [10–13]. Additionally, there were some previous studies to investigate a relation between the robustness and the modularity. For example, it was shown that the modularized structure of bone networks improves the robustness compared to a regular network of the same size [14]. Some other studies observed that both the robustness and the modularity characteristics could be emergently improved through a network evolution process [15, 16]. Moreover, there were some studies to explicitly examine linear correlations between the robustness and the modularity over differently structured networks [17–19]. In metabolic networks, the robustness against the mutant concentrations of metabolites or the mutant expression of enzymes has increased or decreased, respectively, as the modularity increases [17]. On the other hand, the robustness against a gene state perturbation was negatively correlated with the modularity in signaling networks [18, 19]. Although these previous studies found interesting relations between the robustness and the modularity, there are some issues needed to be investigated as follows. The first issue is that there is little known knowledge about changes of the modularity and the robustness. In particular, there was no intensive study about the relationship of the changes of the modularity and the robustness by structural mutations. We note that the previous studies [17–19] focused on the robustness and the modularity over networks with very different structures, whereas this study focuses on the changes of the robustness and the modularity over mutant networks with a slight structural modification. This means that the findings in the previous studies do not necessarily hold in our analysis. Another interesting issue is whether some well-known motifs are relevant to the changes of the modularity and the robustness or not. In fact, some previous studies have shown that network motifs such as feedback loops (FBLs) and feed-forward loops (FFLs) ubiquitously found in various biological networks can affect the robustness [13, 20]. For instance, it was reported that more positive and less negative FBLs are observed in robust networks [21]. Another study showed that coherent coupling of FBLs is a design principle of a robust signaling network [22]. It was also reported that coherent FFLs strengthen the robustness against update-rule perturbations [13]. To our best knowledge, even there was no reported motif which is relevant to the modularity property. Taken together, there is little known about motifs which indicate the changes of the modularity, the robustness, or both. The last issue is that there was no previous study to compare sets of nodes or interactions which efficiently control the changes of the modularity and the robustness. This can be impressive because the result can be used to identify functionally important nodes or interactions such as drug targets.

In this work, we tried to investigate the changes of the modularity and the robustness by edge-removal mutations in signaling networks. Through intensive simulations using a Boolean network model [23, 24], we first found that both the modularity and the robustness increased on average against edge-removal mutations, but the change of modularity is negatively correlated with the change of robustness. More intriguingly, the modularity change was positively correlated with the degree, the number of FBLs, and the edge betweenness of removed edges, whereas the robustness change was negatively correlated with them. Additionally, we found that these findings are consistently conserved in the random networks. Moreover, we identified two groups of genes which are incident to the highly-modularity-increasing and the highly-robustness-decreasing edges against the edge-removal mutations, respectively, and observed that they are likely to be central by forming a considerably large connected component. The gene-ontology enrichment of each of the gene groups was clearly different from the rest of genes. Finally, we found that the highly-robustness-decreasing edges can be promising edgetic drug-targets. Taken together, the analysis of the changes of the robustness and the modularity against the edge-removal mutations can be useful to reveal novel dynamical characteristics of signaling networks.

## Methods

### Network modularity

In this study, we examined the modularity by using the method in a previous study [25] and it has been widely used in many previous studies [18, 19, 26, 27]. Given a directed graph *G*(*V*, *A*) where *V* and *A* denote a set of nodes and a set of directed edges, respectively, we consider a partition *P* = {*V*_1_,  *V*_2_, …,  *V*_*M*_} of *V*(i.e. *V*_*i*_ ∩ *V*_*j*_ = ∅ for all *i* ≠ *j*, and $\bigcup \limits_{i=1}^M{V}_i=V$). The modularity of *P* is defined as $M(P)=\sum \limits_{i=1}^M\left(\frac{\omega_{V_i{V}_i}}{\omega }-\frac{\omega_{V_i}^{in}{\omega}_{V_i}^{out}}{\omega^2}\right)$, where ${\omega}_{V_i{V}_i}$ is the number of directed edges whose both end nodes belong to *V*_*i*_, ${\omega}_{V_i}^{out}$ and ${\omega}_{V_i}^{in}$ are the numbers of directed edges whose starting or ending node only, respectively, belongs to *V*_*i*_, and *ω* is the total number of directed edges in the network. Then the network modularity can be defined as *M*(*G*) = max_*P*_ *M*(*P*). Since it is difficult to optimize the partition, we computed the averaged modularity value of 30 trials of partitions optimized by an existing algorithm [28].

### Boolean network dynamics

In this study, we employed a Boolean network model introduced in previous studies [29, 30] to investigate the complex dynamics of biological networks. In a Boolean network of a directed graph *G*(*V*, *A*), *V* and *A* denote a set of Boolean variables and a set of ordered pairs of the Boolean variables called directed edges, respectively. A state of each *v*_*i*_ ∈ *V* is 1 or 0 which represents on or off state of the gene, respectively. Then a state of a network *G* is defined as a vector of the states of all nodes. A directed edge (*v*_*j*_, *v*_*i*_) ∈ *A* has a positive (activating) or negative (inhibiting) relationship from *v*_*j*_ to *v*_*i*_. Here, we used a nested canalyzing function (NCF) model [31] (see Additional file 1: Supporting Text section for details), which can represent a variety of canalyzing rules in real molecular interactions [32] can be generated by using the NCF model. Additionally, NCFs properly fit the experimental data gained from literature [31], and can also describe logical interaction rules extracted from gene expression experiments [32, 33]. In this study, each NCF is randomly generated by specifying all *I*_*m*_ *s* and *O*_*m*_ *s* between 0 and 1 uniformly at random.

Let *G*(*V*, *A*) a Boolean network with a list of update-rules *F* = {*f*_1_, *f*_2_, …, *f*_*N*_}. Every initial state converges to an attractor which can describe diverse network dynamics such as multi-stability, homeostasis, and oscillation [34, 35]. Let *α*(*s*, *G*, *F*) the attractor which the initial state *s* converged. The network is considered as robust against a perturbation at *v*_*i*_ if the attractor is conserved and we herein considered an update-rule mutation which describes a scenario that *F* is changed to ${F}_i^{\prime }=\left\{{f}_1,\dots, {f}_i^{\prime },\dots, {f}_N\right\}$, where ${f}_i^{\prime }$ means that every canalyzing and canalyzed values were flipped (i.e., all *I*_*m*_ and *O*_*m*_ are changed into 1 − *I*_*m*_ and 1 − *O*_*m*_, respectively). This update-rule mutation may represent a deleterious change in the function of a protein or gene [36], and have been used in a previous study [13]. Then the network robustness *γ*(*G*) is defined as follows:

$$ \gamma (G)=\frac{1}{N\left|S\right|}\sum \limits_{s\in S}\sum \limits_{i=1}^NI\left(\alpha \left(s,G,F\right)=\alpha \left(s,G,{F}_i^{\prime}\right)\right), $$

where *S* is a set of initial states (i.e. *S* = 2^*N*^), and *I*(∙) is a function which outputs 1 or 0 if the condition is met or not, respectively. Because ∣*S*∣ is a very large number, we used a sample subset $\overset{\sim }{S}\subseteq S$ with $\left|\overset{\sim }{S}\right|=2N$ instead of *S* to calculate *γ*(*G*).

### Changes of modularity and robustness by edge-removal mutations

**Fig. 1:**
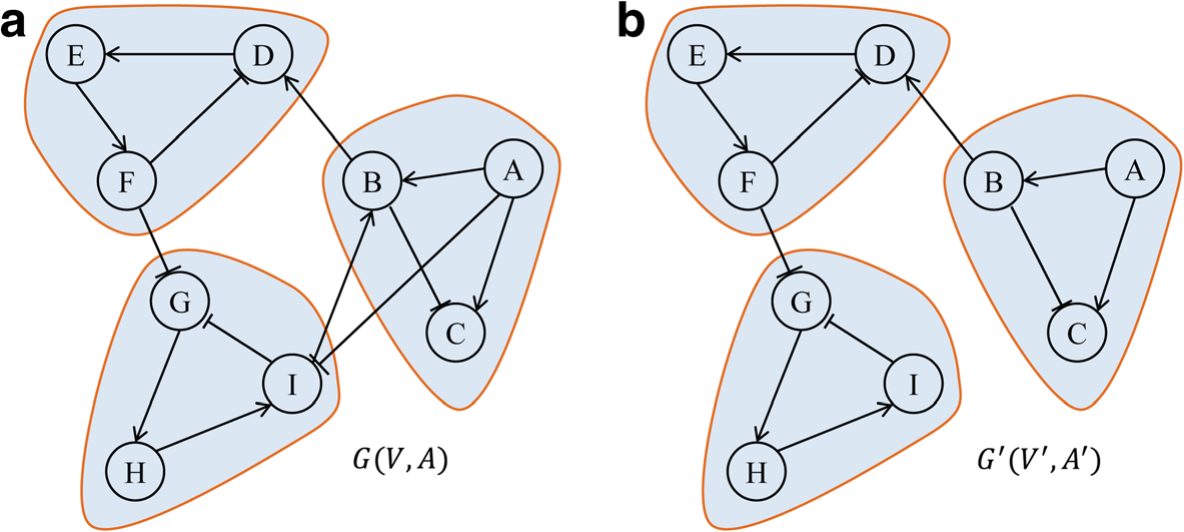
An illustrative example of edge-removal mutations. **a** The original network *G*(*V*, *A*). **b** The mutant network *G* ^′^(*V*, *A* ^′^) by removal of *I* → *B* and *A* ⊣ *I*. It was observed that both networks *G* and *G* ^′^ consist of three modules. Modularity and robustness values in *G* were 0.35799 and 0.88889, respectively, whereas those in *G* ^′^ were 0.48347 and 0.74444, respectively. Therefore, the changes of the modularity and the robustness were positive (0.12548) and negative (−0.14445), respectively

This study focuses on how the modularity and the robustness of a network are changed by edge-removal mutations. Let *m*_1_ and *r*_1_ be the modularity and the robustness of the wild-type network, respectively. Given a removal rate parameter *n* (%), the mutant network is constructed by simultaneously removing approximately *n* percent of a total number of edges from the wild-type network. Then let *m*_2_ and *r*_2_ be the modularity and the robustness of the mutant network. We defined the changes of the modularity and the robustness by the edge-removal mutations as (*m*_2_ − *m*_1_) and (*r*_2_ − *r*_1_), respectively. An illustrative example of the notion about the changes of modularity and robustness by edge-removal mutations is shown in Fig. 1.

### Signaling network datasets

To investigate real signaling networks, we used three datasets of signaling networks: a T-LGL survival network (T-LGL) [37] consisting of 60 genes and 142 interactions, a signal transduction network in fibroblasts (STF) [38] consisting of 139 genes and 557 interactions, and a HIV-1 interaction network in T-cell (HIV-1) [39] consisting of 138 genes and 368 interactions collected by manually curating signaling pathways from cellcollective (www.cellcollective.org) [40].

### Generation of interaction-shuffled random networks

We need to extensively simulate randomly structure networks to verify that the new findings in real networks are generally conserved. In this study, we employed a shuffling model to generate random networks [10, 18]. Given a reference network, it rewires some edges in a way that in-degree and out-degree of every node are conserved. Accordingly, the structure of the generated random network is considerably similar to that of the original network.

### Edge-based structural properties

A previous study has reported that there exists a relationship between a structural property with respect to genes or interactions and the global stability in biological networks [41]. In this regard, we investigated the relations of the following edge-based structural characteristics to the changes of the modularity and the robustness.

- Degree of a node means the number of links incident to the node in a graph. On the other hand, the degree of an interaction (*DEG*) means the sum of the degrees of both end nodes of the edge.
- An FBL is a circular chain where nodes are not revisited except the starting and the ending nodes [42]. It plays an important role in controlling the dynamical behaviors of cellular signaling networks. Specifically, *v*_0_ → *v*_1_ → *v*_2_ → … → *v*_*L* − 1_ → *v*_*L*_ is an FBL of length *L* (≥1) if there exist links from *v*_*i* − 1_ to *v*_*i*_ (*i* = 1, 2, …, *L*) with *v*_0_ = *v*_*L*_ and *v*_*j*_ ≠ *v*_*k*_ for *j*, *k* ∈ {0, 1, …, *L* − 1} and *j* ≠ *k*. The number of FBLs of a link *e* denoted by *NuFBL*(*e*) means the number of different FBLs involving *e*.
- Edge Betweenness (*EBEW*) is defined as the number of shortest paths between pairs of nodes that run along an edge [2], similar to Betweenness of a node. EBEW has been used as an important edge-based centrality measure in a previous study [43].

### Software for statistical tests

In this study, IBM SPSS statistics [44] was used to conduct all statistical tests.

## Results and Discussion

### Relationship between changes of modularity and robustness by edge-removal mutations

**Fig. 2:**
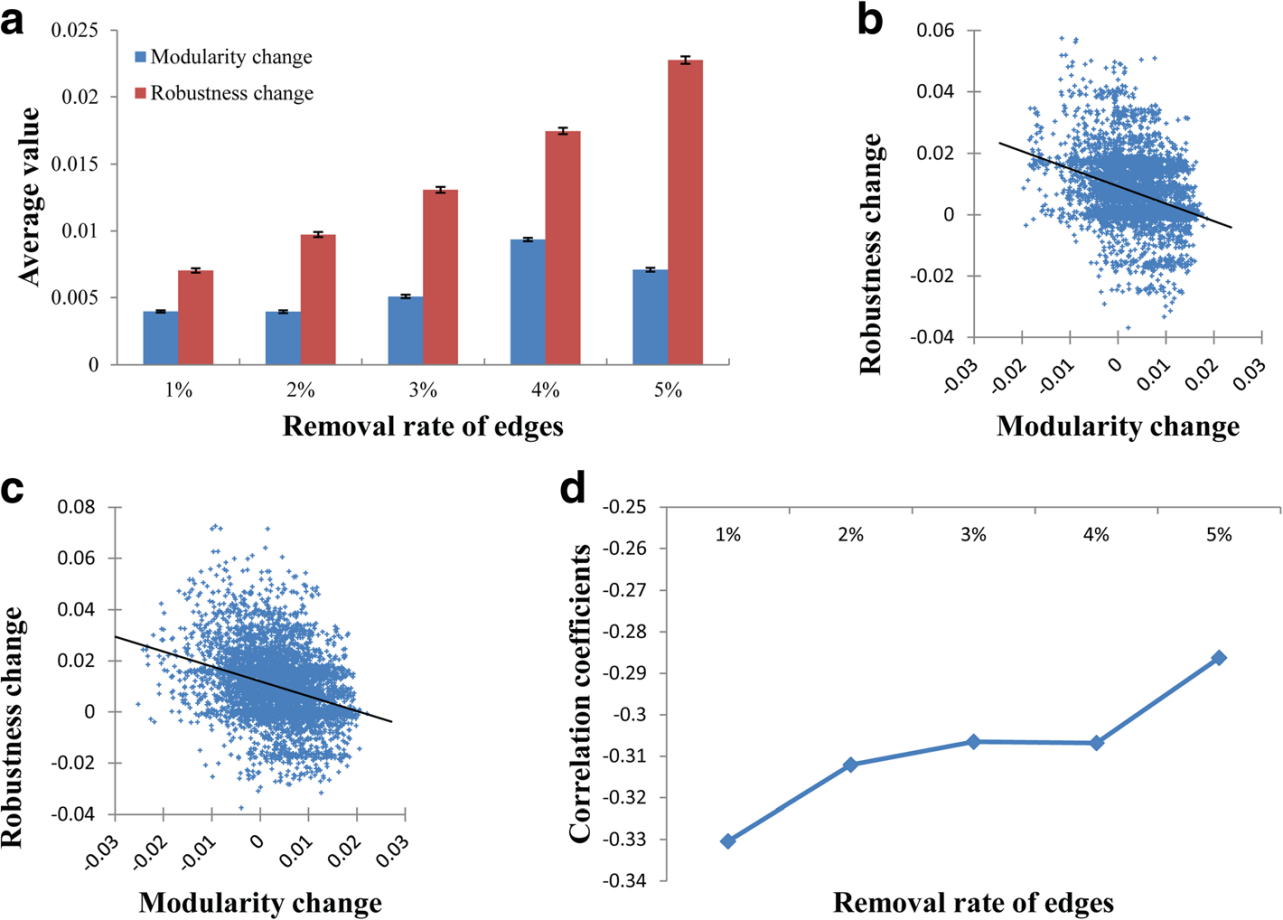
Analysis of the changes of the modularity and the robustness by edge-removal mutations in T-LGL signaling network. The removal rate of edges was varied from 1% to 5% (More specifically, the numbers of removed edges were 2, 3, 4, 5, and 7, respectively, among a total of 142 edges). For each removal rate, 5000 trials of edge-removal were examined. See Additional file 1: Fig. S1 and S2 for the analysis results of STF and HIV-1 signaling networks. **a** Results of average changes of the modularity and the robustness against the removal rate of edges. Y-axis value and error bar represents the average and the standard deviation divided by the square root of the sample size (5000), respectively. Both average values were significantly larger than zero (All *P*-values <0.0001 using one-sample t-test). The one-sample t-test was valid because the average values were normally distributed (see Additional file 1: Fig. S3) and there were no or very few significant outliers (see Additional file 1: Fig. S6). **b-c** Relationship between the changes of the modularity and the robustness in the case that the removal rate is 1% and 2%, respectively. A significant negative relationship was observed (Correlation coefficients were −0.33042 and −0.31208 in (**b**) and (**c**), respectively, with all *P*-values <0.0001). This relationship was consistently observed for larger removal rates (see Additional file 1: Fig. S9). **d** A trend of correlation coefficients between the changes of the modularity and the robustness against the removal rate of edges

We first investigated the changes of the modularity and the robustness by edge-removal mutations in three real networks T-LGL, STF, and HIV-1 (see Methods), and the results are shown in Fig. 2. (T-LGL) and Fig. S1-S2 (STF and HIV-1, respectively) in Additional file 1. In this study, we computed the average changes of the modularity and the robustness values over 5000 trials of edge-removal mutations. In addition, we varied the removal rate, which denotes the percentage of the number of removed edges over the total number of edges, from 1% to 5%. We first tested whether the average changes are significantly positive using one-sample t-test. We note that the average changes were normally distributed, as assessed by Kolmogorov-Smirnov’s test (see Fig. S3-S5 in Additional file 1 for details) and there were no or very few significant outliers, as assessed by a boxplot inspection (see Fig. S6-S8 in Additional file 1 for details). As shown in Fig. 2(a), we observed that both average changes were positive for all removal rates, which means that the modularity and the robustness values were increased by edge-removal (All *P*-values <0.0001; see Additional file 1: Figure S1 (a) and Figure S2 (a) for the results of STF and HIV-1 networks, respectively).

In addition, the increase of the robustness was positively related to the removal rate. To examine the relationship between the changes of modularity and robustness values, we scattered them in the cases that the removal rate is 1% (Fig. 2(b)) and 2% (Fig. 2(c)). Intriguingly, there was a negative correlation between the modularity change and the robustness change, and this was consistently observed in the cases of larger removal rates (see Additional file 1: Figure S9) and the other networks (see Additional file 1: Figure S1 (b)-(c)) and Fig. S2 (b)-(c)). Figure 2 (d) shows the trend of the correlation coefficient values between the changes of modularity and robustness values against the removal rate, and we observed that they were significantly negative irrespective of the removal rates (see Additional file 1: Figure S1 (d) and S2 (d) for the results of STF and HIV-1 networks, respectively). Actually, the negative relationship between the modularity and the robustness in signaling networks was observed in our previous studies [18, 19]. However, it should be noted that the previous finding does not imply any relation between the changes of the modularity and the robustness by edge-removal mutations. To further examine if the negative relationship we found is a general property in randomly structured networks, we generated three sets of 100 random networks shuffled from T-LGL, STF, and HIV-1 (see Methods), and could observe consistent (see Additional file 1: Figure S10). This implies that such the negative relation between the changes of the modularity and the robustness can be regarded as a general principle conserved in randomly structured networks.

### Structural characteristics to affect the changes of the modularity and the robustness

**Fig. 3:**
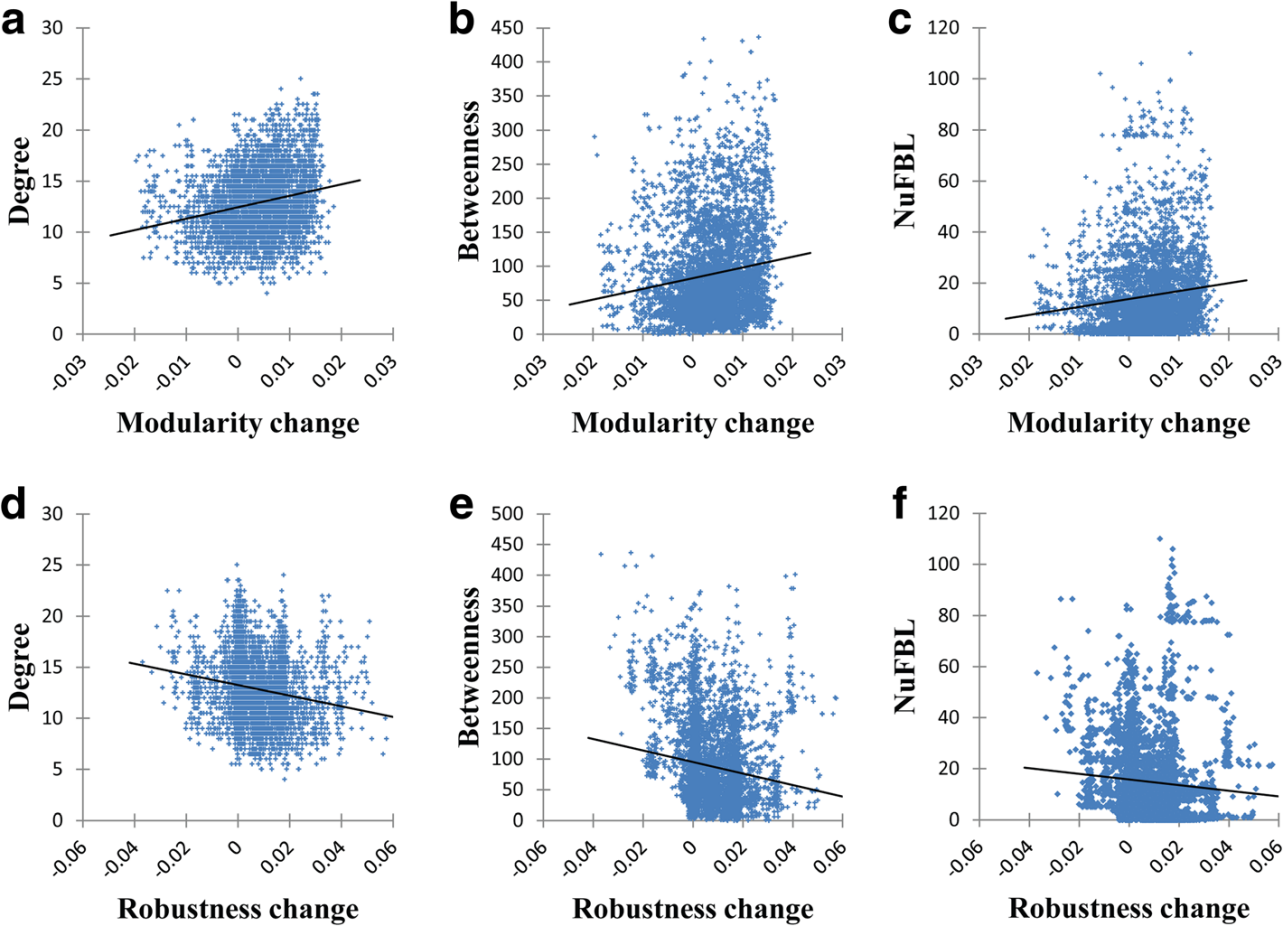
Relationship of each of the changes of the modularity and the robustness with the edge-based structural properties in T-LGL signaling network. The removal rate was set to 1%, and a total of 5000 trials of removals were examined. **a**-**c** Relations of the change of modularity with edge-based degree, EBEW, and NuFBL, respectively. The change of modularity was significantly positively correlated with all structural properties (Correlation coefficients were 0.22443, 0.14564, and 0.12888, respectively, with all *P*-values < 0.0001). **d**-**f** Relations of the change of robustness with edge-based degree, EBEW, and NuFBL, respectively. The change of robustness was significantly negatively correlated with all structural properties (Correlation coefficients were −0.18050, −0.15030, and −0.07933, respectively, with all P-values < 0.0001)

We showed that the changes of the modularity and the robustness are correlated when a network is subject to edge-removal mutations. To reveal structural characteristics to affect the changes of the modularity and the robustness, we investigated the correlations of each of the changes of the modularity and the robustness with each of three edge-based structural properties, DEG, NuFBL and EBEW (see Methods for the definitions) in T-LGL signaling network (Fig. 3; see Additional file 1: Figure S11 and S12 for the results of STF and HIV-1, respectively). In Fig. 3, average DEG, NuFBL, and EBEW values of the removed edges over 5000 trials with 1% of the removal rate were examined. Intriguingly, we found that the change of the modularity is positively correlated with the average DEG, EBEW and NuFBL of removed edges (The correlation coefficients in Fig. 3 (a)-(c) were 0.24708, 0.13786, and 0.11720, respectively, with all *p*-values < 0.001). That is to say, removing edges with a higher degree, EBEW, or NuFBL is more likely to increase the network modularity. These results can be relevant to previous results. For example, the edges with high betweenness values are most likely to lie between subgraphs [45], and thus removing those edges could make a network more separately or more modularized. We also found that the change of the robustness is negatively correlated with the average DEG, EBEW and NuFBL of the removed edges (The correlation coefficients in Fig. 3(d)-(f) were −0.21738, −0.14694, and −0.10537, respectively, with all p-values < 0.0001). In other words, removing edges with a higher degree, EBEW, or NuFBL is more likely to decrease the network robustness. These results can be compared with some previously known results regarding node-based mutations. For example, some studies reported that a node involving more FBLs is likely to be sensitive against node-based mutations. To show that these results hold in random networks, we generated three sets of 100 random Boolean networks each of which was shuffled from T-LGL, STF, and HIV-1 networks, respectively. Through extensive simulations with the removal rate of 1%, we could observe consistent results (see Additional file 1: Fig. S13-S15, All *P*-values < 0.0001 using t-test). In other words, the degree, the edge-betweenness and the number of FBLs were positively correlated with the change of the modularity whereas they were negatively correlated in the random networks. It means that those structural characteristics might be a vital factor in controlling both the changes of the modularity and the robustness.

### Topological distribution of highly modularity-increasing and robustness-decreasing edges by removal mutations

**Fig. 4:**
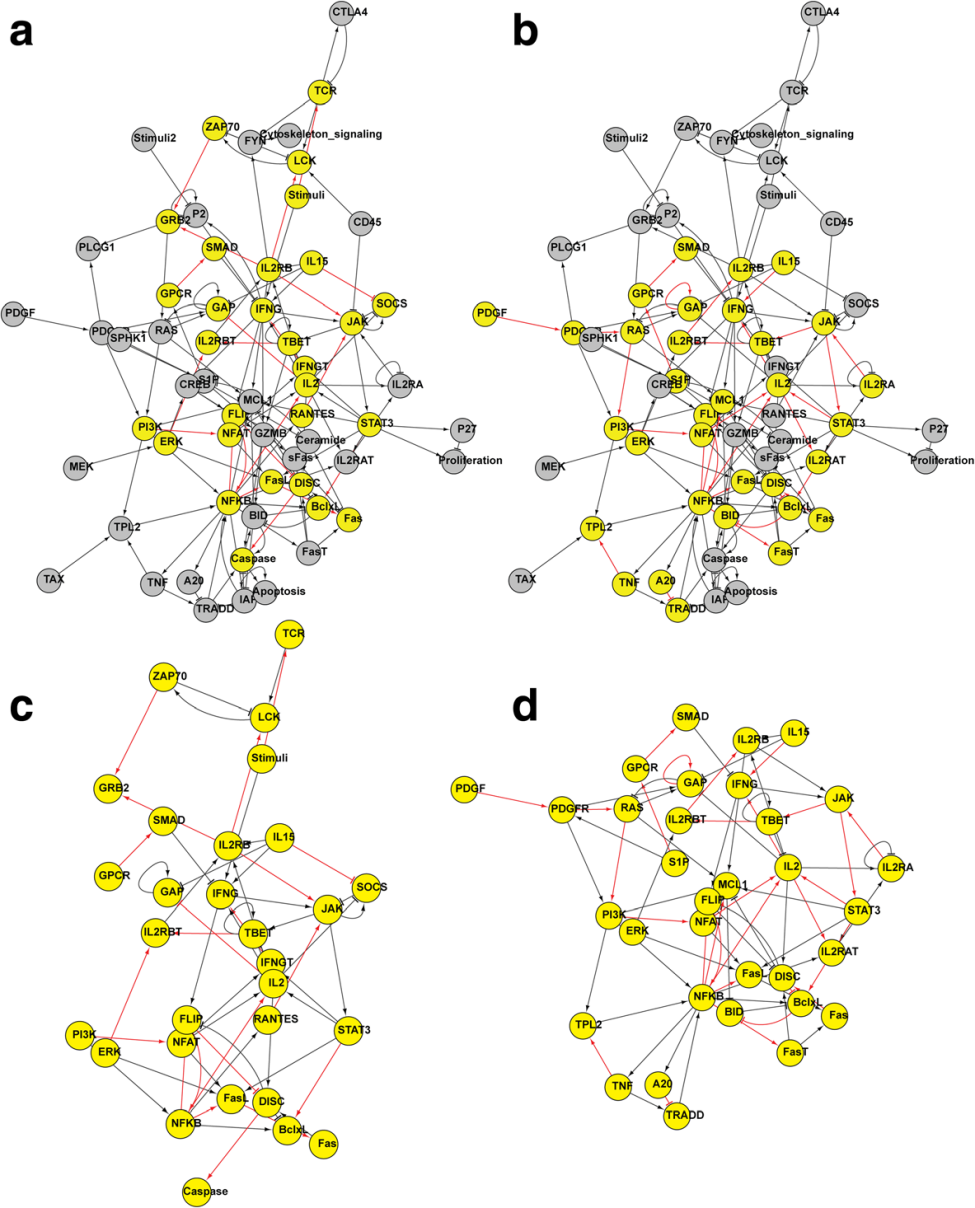
Topological distributions of High-MI/High-RD edges and their incident nodes in T-LGL signaling network. **a**-**b** Distributions of High-MI and High-RD edges, respectively, and their incident nodes. **c**-**d** Subgraphs with respect to High-MI-incident and High-RD-incident nodes, respectively. Red link and yellow node represent High-MI edge and High-MI-incident node, respectively, in both (**a**) and (**c**), whereas they represent High-RD edge and High-RD-incident node, respectively, in both (**b**) and (**d**). (see Additional file 1: Fig. S16-S17 for the results of STF and HIV-1 networks.)

**Fig. 5:**
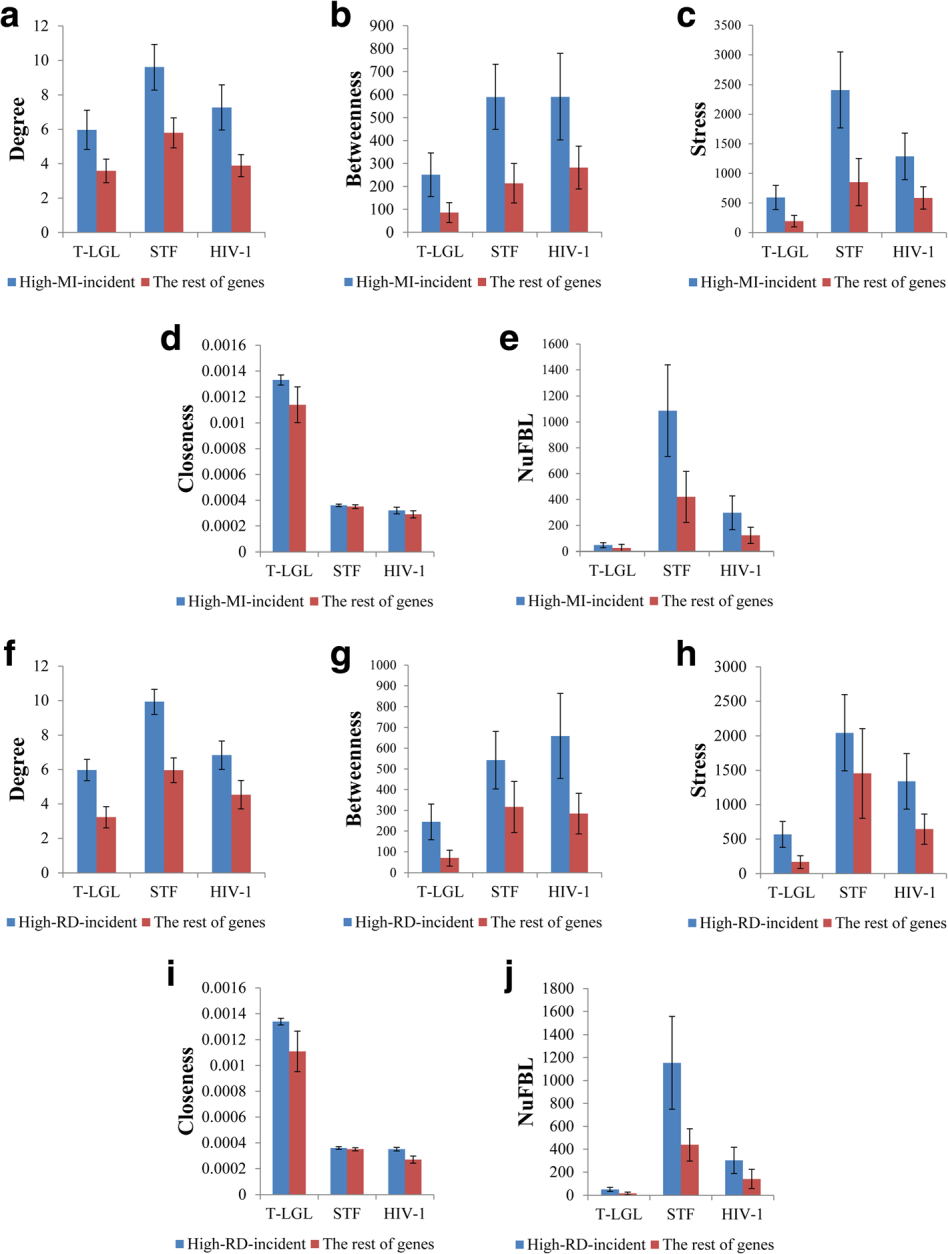
Comparison of node-based centralities between High-MI-incident/High-RD-incident group and the rest of genes in the signaling networks. **a-e** Results of node-based degree, betweenness, stress, closeness, and NuFBL, respectively, with respect to High-MI-incident group. In each subfigure, “The rest of genes” means non “High-MI-incident” genes. **f-j** Results of node-based degree, betweenness, stress, closeness, and NuFBL, respectively, with respect to High-RD-incident group. In each subfigure, “The rest of genes” means non “High-RD-incident” genes. For all subfigures, Y-Axis value and error bar represents the average and 95% confidence interval, respectively. Genes belonging to High-MI-incident and High-RD-incident group showed higher degree, node-based betweenness, stress, closeness, and the number of involved feedback loops than the rest of genes (All P-values < 0.05 except stress of STF in (**c**), closeness of HIV-1 in (**d**), and NuFBL of T-LGL in (**j**))

In the previous subsection, it was shown that the change of the modularity is positively correlated with the degree, the edge betweenness, and the number of involved FBLs with respect to the removed edges whereas the change of robustness is negatively correlated with them. From these results, we hypothesized that the edges whose removal will increase the modularity or decrease the robustness tend to be centrally located in signaling networks. To validate this hypothesis, we first specified “Highly-modularity-increasing” (High-MI) and “Highly-robustness-decreasing” (High-RD) sets of edges as follows: We examined the changes of the modularity and the robustness over 5000 trials of edge-removal mutations with 1% removal rate, and collected top-*K* set of edges among them in an increasing (resp. decreasing) order of the change of the modularity (resp. the robustness). Considering the distributions of the change of the modularity (resp. robustness), *K* was chosen to 20, 20, and 18 (resp., 31, 18, and 16) for T-LGL, STF, and HIV-1 networks, respectively. Then High-MI (resp. High-RD) denotes the union of the edges each of which was included in the modularity-increasing (resp. robustness-decreasing) top-*K* edges. Accordingly, we identified High-MI (High-RD) groups consisting of 22, 79, and 42 edges (resp. 30, 69, and 33 edges) in T-LGL, STF, and HIV-1 networks, respectively. Furthermore, we defined High-MI-incident (High-RD-incident) group which is a set of genes incident to an edge in the High-MI (resp. High-RD) edge group, and found the number of genes in the High-MI-incident (resp. High-RD-incident) were 29, 81, and 59 (resp. 33, 72, and 48) in T-LGL, STF, and HIV-1 networks, respectively. The topological distributions of High-MI and High-RD edge sets in T-LGL, STF, and HIV-1 networks are shown in Fig. 4 and Figure S16-S17 in Additional file 1, respectively. As expected, it was observed that the edges in High-MI and High-RD groups are likely to be located at the centre of the signaling network. In order to more clarify this observation, we compared node-based centrality values between each set of High-MI-incident and High-RD-incident groups and the set of rest genes. Specifically, we computed average degree, node-based betweenness [43], stress [46], closeness [47], and the number of involved FBLs [22] for each group of nodes (Fig. 5). As depicted in the figure, we found that genes of High-MI-incident and High-RD-incident groups showed higher degree, node-based betweenness, stress, closeness, and the number of FBLs than the rest of genes (Only three cases among 30 comparisons did not show significant differences.) In other words, the genes incident to the interactions whose greatly increase the modularity or decrease the robustness tends to be central in the signaling network. Additionally, we visualized the connectedness of edges of High-MI and High-RD groups by projecting them into a subnetwork from T-LGL (see Fig. 4(c) and (d), respectively), STF (see Additional file 1: Fig. S16(c) and (d), respectively), and HIV-1 (see Additional file 1: Figure S17(c) and (d), respectively) networks. As shown in the figures, every subnetwork forms a single connected component. This implies that the highly modularity-increasing or robustness-decreasing edges with respect to edge-removal mutations are closely located in signaling networks.

### Gene ontology analysis of a set of genes incident to highly-modularity-increasing or highly-robustness-decreasing edges

We conducted Gene Ontotlogy (GO) enrichment analysis (The Gene Ontotlogy Consortium, 2008) using ClueGO tool [48] to investigate the locational and functional characteristics of sets of High-MI-incident and High-RD-incident genes. The results are shown in Table 1 (see Additional file 1: Table S1-S2). Some GO terms such as protein tyrosine kinase and peptidase activity are more highly observed in High-MI-incident and High-RD-incident groups. The former is an enzyme which transfers a phosphate group from adenosine triphosphate to a protein in a cell, and the latter is catalysis of the hydrolysis of a peptide bond. In addition, High-MI-incident and High-RD-incident gene groups showed a greater fraction of response function terms. Regulation of adaptive immune response is any process that modulates the frequency, rate, or extent of an adaptive immune response regarding to robustness change. Furthermore, High-MI-incident group showed a greater portion of vital binding functions. For example, a protein phosphatase is an enzyme that removes a phosphate group from the phosphorylated amino acid residue of its substrate protein, and its binding function is interacting selectively and non-covalently with any protein phosphatase. On the other hand, High-RD-incident group showed a greater fraction related to signaling pathway. For instance, necroptosis is a programmed form of necrosis, or inflammatory cell death, and its signaling pathway is a series of molecular signals which triggers the necroptotic death of a cell. Taken together, significantly different functions between High-MI-incident/High-RD-incident groups of genes and the rest of genes can be characterized.

**Table 1:** Results of GO analysis between High-MI-incident/High-RD-incident group and the rest of genes in T-LGL signalling network, with all P-values using Bonferroni test (see Additional file 1: Table S1 and S2 for the results of STF and HIV-1 signaling networks)

Type of GO analysis	GO term	High-MI-incident (%)	The rest of genes (%)	*P*-value
Modularity change	Non-membrane spanning protein tyrosine kinase activity	75.00	25.00	5.4E-6
	Positive regulation of peptidase activity	57.62	48.01	3.4E-12
	Positive regulation of adaptive immune response	75.00	25.00	34.0E-6
	Regulation of immunoglobulin mediated immune response	75.00	25.00	2.9E-6
	Phosphatase binding	85.71	14.29	72.0E-9
	Protein phosphatase binding	83.33	16.67	260.0E-9
	Cytokine receptor binding	64.29	35.71	2.1E-15
	Death receptor binding	60.00	40.00	960.0E-12
	GO term	High-RD-incident (%)	The rest of genes (%)	*P*-value
Robustness change	Positive regulation of cysteine-type endopeptidase activity	63.62	42.41	24.0E-12
	Positive regulation of peptidase activity	67.22	38.41	3.4E-12
	Response to nicotine	60.00	40.00	200.0E-9
	Regulation of adaptive immune response	83.33	16.67	540.0E-9
	Positive regulation of apoptotic signaling pathway	67.22	38.41	13.0E-12
	Regulation of NIK/NF-kappaB signaling	75.00	25.00	5.4E-6
	Necroptotic signaling pathway	79.25	26.42	380.0E-9
	Extrinsic apoptotic signaling pathway in absence of ligand	66.84	40.11	370.0E-12

### Edge-based drug discovery

**Fig. 6:**
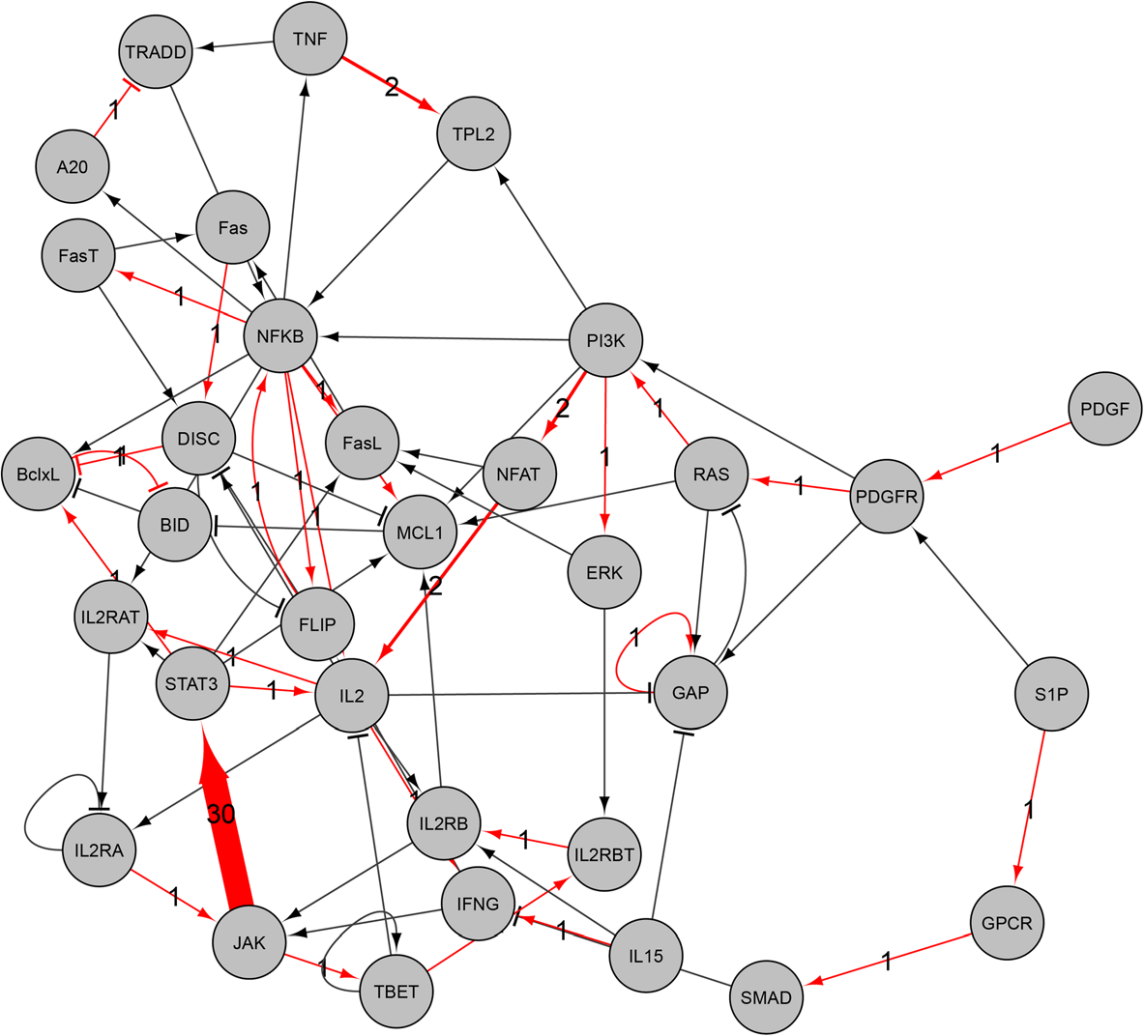
Edge-removal analysis for edgetic drug discovery in T-LGL signaling network. The arrows and bar-headed lines represent positive and negative interactions, respectively. Line thickness is proportional to the inclusion frequency of the interaction in top-*K* edge sets ranked in a decreasing order of the robustness change among 5000 trials of edge-removal mutations with 1% removal rate. The interaction (*JAK* → *STAT*3) was observed 30 times in top-*K* edge sets (*K* was chosen to 30). (see Additional file 1: Fig. S18 and S19 for the results of STF and HIV-1 networks)

We performed a case study to show an application for edge-based drug discovery. For every interaction in High-RD group, we examined the inclusion frequency of the interaction in top-*K* edge sets ranked by a decreasing order of the robustness change among 5000 trials of edge-removal mutations with 1% removal rate. We found that (*JAK* → *STAT*3), (*IP*3*R*1 → *Ca*), and (*gp*41 → *CD*28) showed the highest frequency in the T-LGL, STF, and HIV-1 networks, respectively. We hypothesized that these edges can be candidates of edgetic drug-targets, because they most frequently caused the highest decreasing robustness through removal mutations. To validate this, we surveyed some recent experimental studies. Regarding (*JAK* → *STAT*3) interaction of T-LGL network (Fig. 6), it was shown that the interaction is associated with oncogenesis, proliferation, survival, metastasis, angiogenesis, and immune evasion in gastrointestinal cancers [49, 50]. For example, a colorectal cancer might be developed by dysregulation of the interleukin (IL)-6-mediated *JAK* → *STAT*3 pathway, and therefore strategies targeting the IL-6/JAK/STAT3 pathway have emerged as attractive options to treat colorectal cancer [51]. Next, the (*IP*3*R*1 → *Ca*) interaction of STF network (see Additional file 1: Fig. S18) played an important role of dynamical relationship between IP3R1 and PI3K, which are the most influential components associated with drug resistance [52]. Systemic analysis of these components and their upstream components has resulted in identifying novel combinations of drug targets. In HIV-1 network, (*gp*41 → *CD*28) was found to be the highest frequency interaction (see Additional file 1: Figure S19), but there was no relevant experimental study to support it. However, we could find biological evidence related to the second highest frequency interaction, (*PI*3*K* → *PIP*3). It is included in PI3K/Akt/mTOR pathway [53], which is known to be frequently activated in ovarian cancer. Therefore, inhibitors targeting this pathway can be evaluated as treatment strategies for ovarian cancer, either mono-therapy or in combination with cytotoxic agents [54]. Another interesting point was the feedback loops involved with those interactions. We found a large number of feedback loops were related to (*JAK* → *STAT*3), (*IP*3*R*1 → *Ca*), and (*PI*3*K* → *PIP*3) in T-LGL, STF, HIV-1, respectively (The numbers were 70, 286, and 872, respectively). Considering that the number of involved FBLs was shown to be associated with the functional importance of a node or an interaction, it implies that the found interactions can be promising drug-targets.

## Conclusions

There have been many computational studies about the network robustness and modularity, whereas there are few studies on investigating the modularity change and the robustness change. Through extensive simulations, we found that both the modularity and the robustness increased on average in mutant networks by edge-removal mutations in this study. However, it was interesting that the changes of the modularity and the robustness were negatively correlated. Another interesting finding is that the changes of the modularity and the robustness are positively and negatively, respectively, correlated with each of the degree, the number of FBLs, and the edge betweenness of removed edges. These results were consistently observed in randomly structure networks. Additionally, we identified two sets of genes which are incident to the highly-modularity-increasing and the highly-robustness-decreasing edges, respectively, and observed that they are likely to be central by forming a large connected component. These two gene sets were enriched with different GO terms and the investigation on the reason why such GO terms are related to modularity and robustness will be a future study. Finally, we found that the highly-robustness-decreasing edge can be considered for promising edge-based drug-targets. Taken together, our results in this study can be useful to unravel novel dynamical characteristics of signaling networks.

## Additional file

Additional file 1: Figure S1. Analysis of the changes of the modularity and the robustness by edge-removal mutations in STF signaling network. **Figure S2.** Analysis of the changes of the modularity and the robustness by edge-removal mutations in HIV-1 signaling network. **Figure S3.** Analysis of normal distributions of averages of modularity changes and robustness changes in T-LGL network. **Figure S4.** Analysis of normal distributions of averages of modularity changes and robustness changes in STF network. **Figure S5.** Analysis of normal distributions of averages of modularity changes and robustness changes in HIV-1 network. **Figure S6.** Analysis of outliers of averages of modularity changes and robustness changes in T-LGL network. **Figure S7.** Analysis of outliers of averages of modularity changes and robustness changes in STF network. **Figure S8.** Analysis of outliers of averages of modularity changes and robustness changes in HIV-1 network. **Figure S9.** Relationship between the changes of the modularity and the robustness in T-LGL signaling network. **Figure S10.** Relationship between the changes of the modularity and the robustness in random networks. **Figure S11.** Relationship of each of the changes of the modularity and the robustness with the structural properties in STF signaling network. **Figure S12.** Relationship of each of the changes of the modularity and the robustness with the structural properties in HIV-1 signaling network. **Figure S13.** Relationship of each of the changes of the modularity and the robustness with the structural properties in random networks shuffled from T-LGL network. **Figure S14.** Relationship of each of the changes of the modularity and the robustness with the structural properties in random networks shuffled from STF network. **Figure S15.** Relationship of each of the changes of the modularity and the robustness with the structural properties in random networks shuffled from HIV-1 network. **Figure S16.** Topological distributions of High-MI/High-RD edges and their incident nodes in STF signaling network. **Figure S17.** Topological distributions of High-MI/High-RD edges and their incident nodes in HIV-1 signaling network. **Figure S18**. Edge-removal analysis for edgetic drug discovery in STF signaling network. **Figure S19**. Edge-removal analysis for edgetic drug discovery in HIV-1 signaling network. **Table S1**. GO analysis results between High-MI-incident/High-RD-incident group and the rest of genes in STF network. **Table S2.** GO analysis results between High-MI-incident/High-RD-incident group and the rest of genes in HIV-1 network. (PDF 3490 kb)

## Abbreviations

DEG: Degree
EBEW: Edge Betweenness
FBLs: Feedback loops
FFLs: Feed-forward loops
GO: Gene ontology

## References

[1] Kitano H (2004). Biological robustness. Nat Rev Genet.

[2] Girvan M, Newman MEJ (2002). Community structure in social and biological networks. Proc Natl Acad Sci.

[3] Ingolia NT (2004). Topology and robustness in the drosophila segment polarity network. PLoS Biol.

[4] Yi T-M, Huang Y, Simon MI, Doyle J (2000). Robust perfect adaptation in bacterial chemotaxis through integral feedback control. Proc Natl Acad Sci.

[5] Little JW, Shepley DP, Wert DW (1999). Robustness of a gene regulatory circuit. EMBO J.

[6] Kreimer A, Borenstein E, Gophna U, Ruppin E (2008). The evolution of modularity in bacterial metabolic networks. Proc Natl Acad Sci.

[7] Lin Y-S, Hsu W-L, Hwang J-K, Li W-H (2007). Proportion of solvent-exposed amino acids in a protein and rate of protein evolution. Mol Biol Evol.

[8] von Dassow G, Munro E (1999). Modularity in animal development and evolution: elements of a conceptual framework for EvoDevo. J Exp Zool.

[9] Han J-DJ, Bertin N, Hao T, Goldberg DS, Berriz GF, Zhang LV, Dupuy D, Walhout AJM, Cusick ME, Roth FP (2004). Evidence for dynamically organized modularity in the yeast protein-protein interaction network. Nature.

[10] Trinh H-C, Kwon Y-K (2016). Edge-based sensitivity analysis of signaling networks by using Boolean dynamics. Bioinformatics.

[11] Paroni A, Graudenzi A, Caravagna G, Damiani C, Mauri G, Antoniotti M (2016). CABeRNET: a Cytoscape app for augmented Boolean models of gene regulatory NETworks. BMC Bioinformatics.

[12] Kaneko K (2007). Evolution of robustness to noise and mutation in gene expression dynamics. PLoS One.

[13] Le D-H, Kwon Y-K (2013). A coherent feedforward loop design principle to sustain robustness of biological networks. Bioinformatics.

[14] Viana MP, Tanck E, Beletti ME (2009). Costa LdF: modularity and robustness of bone networks. Mol BioSyst.

[15] Variano EA, McCoy JH, Lipson H (2004). Networks, dynamics, and modularity. Phys Rev Lett.

[16] Hintze A, Adami C (2008). Evolution of complex modular biological networks. PLoS Comput Biol.

[17] Holme P (2011). Metabolic robustness and network modularity: a model study. PLoS One.

[18] Truong C-D, Tran T-D, Kwon Y-K (2016). MORO: a Cytoscape app for relationship analysis between modularity and robustness in large-scale biological networks. BMC Syst Biol.

[19] Tran T-D, Kwon Y-K (2013). The relationship between modularity and robustness in signalling networks. J R Soc Interface.

[20] Kim J-R, Yoon Y, Cho K-H (2008). Coupled feedback loops form dynamic motifs of cellular networks. Biophys J.

[21] Kwon Y-K, Cho K-H (2008). Quantitative analysis of robustness and fragility in biological networks based on feedback dynamics. Bioinformatics.

[22] Kwon Y-K, Cho K-H (2008). Coherent coupling of feedback loops: a design principle of cell signaling networks. Bioinformatics.

[23] Kauffman S (2004). A proposal for using the ensemble approach to understand genetic regulatory networks. J Theor Biol.

[24] Graudenzi A, Serra R, Villani M, Colacci A, Kauffman SA (2011). Robustness analysis of a Boolean model of gene regulatory network with memory. J Comput Biol.

[25] Leicht EA, Newman MEJ (2008). Community structure in directed networks. Phys Rev Lett.

[26] Fortunato S (2010). Community detection in graphs. Phys Rep.

[27] Mucha PJ, Richardson T, Macon K, Porter MA, Onnela J-P (2010). Community structure in time-dependent, multiscale, and multiplex networks. Science.

[28] Noack A (2009). Modularity clustering is force-directed layout. Phys Rev E.

[29] Campbell C, Albert R (2014). Stabilization of perturbed Boolean network attractors through compensatory interactions. BMC Syst Biol.

[30] Steinway SN, Biggs MB, Loughran TP, Papin JA, Albert R (2015). Inference of network dynamics and metabolic interactions in the gut microbiome. PLoS Comput Biol.

[31] Kauffman S, Peterson C, Samuelsson B, Troein C (2003). Random Boolean network models and the yeast transcriptional network. Proc Natl Acad Sci.

[32] Harris SE, Sawhill BK, Wuensche A, Kauffman S (2002). A model of transcriptional regulatory networks based on biases in the observed regulation rules. Complexity.

[33] Naldi A, Carneiro J, Chaouiya C, Thieffry D (2010). Diversity and plasticity of Th cell types predicted from regulatory network Modelling. PLoS Comput Biol.

[34] Bhalla US, Ram PT, Iyengar R, Kinase Phosphatase MAP (2002). As a locus of flexibility in a mitogen-activated protein kinase signaling network. Science.

[35] Pomerening JR, Sontag ED, Ferrell JE (2003). Building a cell cycle oscillator: hysteresis and bistability in the activation of Cdc2. Nat Cell Biol.

[36] Ng PC, Henikoff S (2003). SIFT: predicting amino acid changes that affect protein function. Nucleic Acids Res.

[37] Saadatpour A, Wang R-S, Liao A, Liu X, Loughran TP, Albert I, Albert R (2011). Dynamical and structural analysis of a T cell survival network identifies novel candidate therapeutic targets for large granular lymphocyte leukemia. PLoS Comput Biol.

[38] Hirabayashi T, Murayama T, Shimizu T (2004). Regulatory mechanism and physiological role of cytosolic phospholipase A_2_. Biol Pharm Bull.

[39] Oyeyemi OJ, Davies O, Robertson DL, Schwartz J-M (2015). A logical model of HIV-1 interactions with the T-cell activation signalling pathway. Bioinformatics.

[40] Helikar T, Kowal B, McClenathan S, Bruckner M, Rowley T, Madrahimov A, Wicks B, Shrestha M, Limbu K, Rogers JA (2012). The cell collective: toward an open and collaborative approach to systems biology. BMC Syst Biol.

[41] Kaiser M, Hilgetag CC (2004). Edge vulnerability in neural and metabolic networks. Biol Cybern.

[42] Ananthasubramaniam B, Herzel H (2014). Positive feedback promotes oscillations in negative feedback loops. PLoS One.

[43] Freeman LC, Set A (1977). Of measures of centrality based on Betweenness. Sociometry.

[44] IBM SPSS Statistics. https://www.ibm.com/products/spss-statistics. Accessed 11 Sept 2017.

[45] Yoon J, Blumer A, Lee K (2006). An algorithm for modularity analysis of directed and weighted biological networks based on edge-betweenness centrality. Bioinformatics.

[46] Shimbel A (1953). Structural parameters of communication networks. The bulletin of mathematical biophysics.

[47] Wuchty S, Stadler PF (2003). Centers of complex networks. J Theor Biol.

[48] Bindea G, Mlecnik B, Hackl H, Charoentong P, Tosolini M, Kirilovsky A, Fridman W-H, Pagès F, Trajanoski Z, Galon J (2009). ClueGO: a Cytoscape plug-in to decipher functionally grouped gene ontology and pathway annotation networks. Bioinformatics.

[49] Nikolaou K, Sarris M, Talianidis I (2013). Molecular pathways: the complex roles of inflammation pathways in the development and treatment of liver cancer. Clin Cancer Res.

[50] Bournazou E, Bromberg J (2013). Targeting the tumor microenvironment: JAK-STAT3 signaling. JAK-STAT.

[51] Wang SW, Sun YM (2014). The IL-6/JAK/STAT3 pathway: potential therapeutic strategies in treating colorectal cancer (review). Int J Oncol.

[52] Puniya BL, Allen L, Hochfelder C, Majumder M, Helikar T (2016). Systems perturbation analysis of a large-scale signal transduction model reveals potentially influential candidates for cancer therapeutics. Frontiers in Bioengineering and Biotechnology.

[53] Slomovitz BM, Coleman RL (2012). The PI3K/AKT/mTOR pathway as a therapeutic target in endometrial cancer. Clin Cancer Res.

[54] Mabuchi S, Kuroda H, Takahashi R, Sasano T (2015). The PI3K/AKT/mTOR pathway as a therapeutic target in ovarian cancer. Gynecol Oncol.

