# Supplementary material for: Investigation on changes of modularity and robustness by edge-removal mutations in signaling networks
Source: BMC Syst Biol. 2017 Dec 21;11(Suppl 7):125. doi: 10.1186/s12918-017-0505-2 (PMC5763305; doi:10.1186/s12918-017-0505-2)
Supplement: Additional file 1: Figure S1. — Analysis of the changes of the modularity and the robustness by edge-removal mutations in STF signaling network. Figure S2. Analysis of the changes of the modularity and the robustness by edge-removal mutations in HIV-1 signaling network. Figure S3. Analysis of normal distributions of averages of modularity changes and robustness changes in T-LGL network. Figure S4. Analysis of normal distributions of averages of modularity changes and robustness changes in STF network. Figure S5. Analysis of normal distributions of averages of modularity changes and robustness changes in HIV-1 network. Figure S6. Analysis of outliers of averages of modularity changes and robustness changes in T-LGL network. Figure S7. Analysis of outliers of averages of modularity changes and robustness changes in STF network. Figure S8. Analysis of outliers of averages of modularity changes and robustness changes in HIV-1 network. Figure S9. Relationship between the changes of the modularity and the robustness in T-LGL signaling network. Figure S10. Relationship between the changes of the modularity and the robustness in random networks. Figure S11. Relationship of each of the changes of the modularity and the robustness with the structural properties in STF signaling network. Figure S12. Relationship of each of the changes of the modularity and the robustness with the structural properties in HIV-1 signaling network. Figure S13. Relationship of each of the changes of the modularity and the robustness with the structural properties in random networks shuffled from T-LGL network. Figure S14. Relationship of each of the changes of the modularity and the robustness with the structural properties in random networks shuffled from STF network. Figure S15. Relationship of each of the changes of the modularity and the robustness with the structural properties in random networks shuffled from HIV-1 network. Figure S16. Topological distributions of High-MI/High-RD edges and their incident nodes in STF signa [file 12918_2017_505_MOESM1_ESM.pdf]

Supplementary Information

Supporting Text. Nested Canalyzing Functions

Given a Boolean network $G(V, A)$, the value of each variable v_i at time $t+1$ is determined by the values of k_i other variables $v_{i_1}, v_{i_2}, \dots, v_{i_{k_i}}$ with a link to v_i at time t by the Boolean function f_i: $v_i(t+1) = f_i(v_{i_1}(t), v_{i_2}(t), \dots, v_{i_{k_i}}(t))$. The rule f_i is called *canalyzing* on the input variable v_{i_m} if there exist Boolean values, I_m and O_m, such that

$$v_{i_m}(t) = I_m \rightarrow v_i(t+1) = O_m.$$

Then, I_m and O_m are called the canalyzing and canalyzed values for the output variable v_i, respectively.

The notion of nested canalyzing functions (NCFs) was introduced in (Kauffman, et al., 2003), and they are a natural subset of canalyzing rules. It was inspired by the question of what happens in the noncanalyzing case: When a rule is not canalyzed by the value of the first input variable, is it canalyzed by one of the remaining input variables? This consecutive canalization test can be repeated for all inputs and therefore an NCFs to update v_i can be represented as follows:

$$v_i(t+1) = \begin{cases} O_1 & if\ v_{i_1}(t) = I_1 \\ O_2 & if\ v_{i_1}(t) \neq I_1 \text{ and } v_{i_2}(t) = I_2 \\ O_3 & if\ v_{i_1}(t) \neq I_1 \text{ and } v_{i_2}(t) \neq I_2 \text{ and } v_{i_3}(t) = I_3 \\ & \vdots \\ O_{k_i} & if\ v_{i_1}(t) \neq I_1 \text{ and } \cdots \text{and } v_{i_{k_i-1}}(t) \neq I_{k_i-1} \text{ and } v_{i_{k_i}}(t) = I_{k_i} \\ 1 - O_{k_i} & otherwise \end{cases}$$

Where all I_m and O_m $(m = 1, 2, \dots, k_i)$ denote the canalyzing and canalyzed values, respectively, and $O_{default} \neq O_{k_i}$. In addition, as in the previous studies (Kauffman, et al., 2003; Kauffman, et al., 2004), we independently and randomly specified $O_1, \dots, O_{k_i}$ values with the probabilities

$$P(O_m = 1) = \frac{\exp(-2^{-m}\theta)}{1 + \exp(-2^{-m}\theta)}$$

where θ is a constant. On the other hand, the value of I_m is deterministically specified by the value of O_m and the sign of the interaction from v_{i_m} to v_i $(m = 1, \dots, k_i)$ as the following table.

O_m	Sign of the interaction from v_{i_m} to v_i	I_m
1	Positive ($v_{i_m} \rightarrow v_i$)	1
1	Negative ($v_{i_m} \dashv v_i$)	0
0	Positive ($v_{i_m} \rightarrow v_i$)	0
0	Negative ($v_{i_m} \dashv v_i$)	1

Supplementary Figures

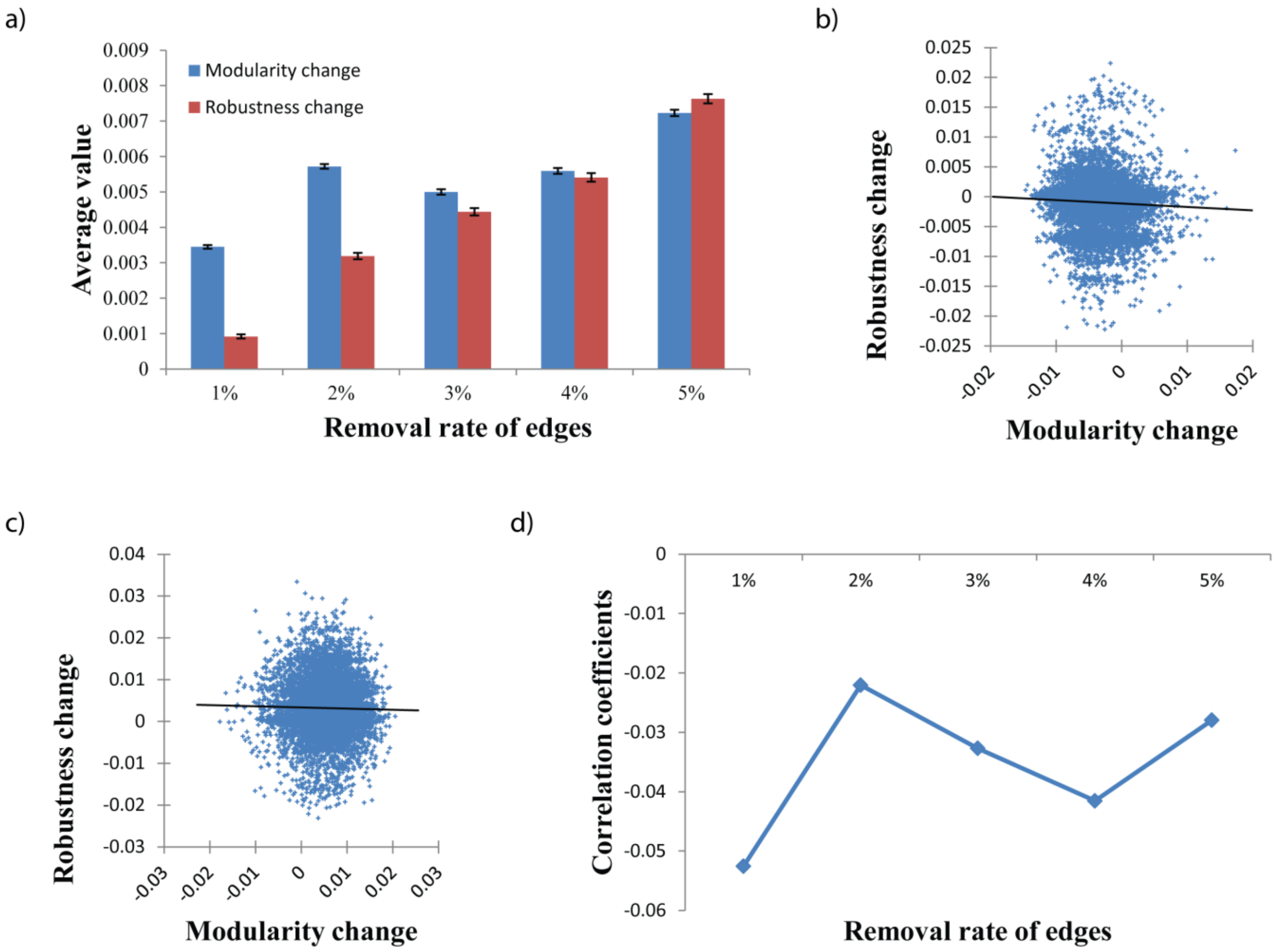

Fig. S1. Analysis of the changes of the modularity and the robustness by edge-removal mutations in STF signaling network. The removal rate of edges was varied from 1% to 5% (More specifically, the numbers of removed edges were 5, 11, 16, 22, and 27, respectively, among a total of 557 edges). For each removal rate, 5,000 trials of edge-removal were examined. **(a)** Results of average changes of the modularity and the robustness against the removal rate of edges. Y-axis value and error bar represent the average and the standard deviation divided by the square root of the sample size (5000), respectively. Both average values were significantly larger than zero (All P-values <0.0001, using one-sample t-test). The one-sample t-test was valid because the average values were normally distributed (see Additional file 1: Fig. S4) and there were very few outliers (see Additional file 1: Fig. S7). **(b)-(c)** Relationship between the changes of the modularity and the robustness in the case that the removal rate is 1% and 2%, respectively. A significant negative relationship was observed (Correlation coefficients were -0.05254 and -0.022068, respectively, with all P-values <0.0001). This relationship was consistently observed for larger removal rates (Correlation coefficients when the removal rate of edge is 3%, 4%, and 5% were -0.03272 and, -0.04156, and -0.02795, respectively, with all P-values <0.0001). **(d)** A trend of correlation coefficients between the changes of the modularity and the robustness against the removal rate of edges.

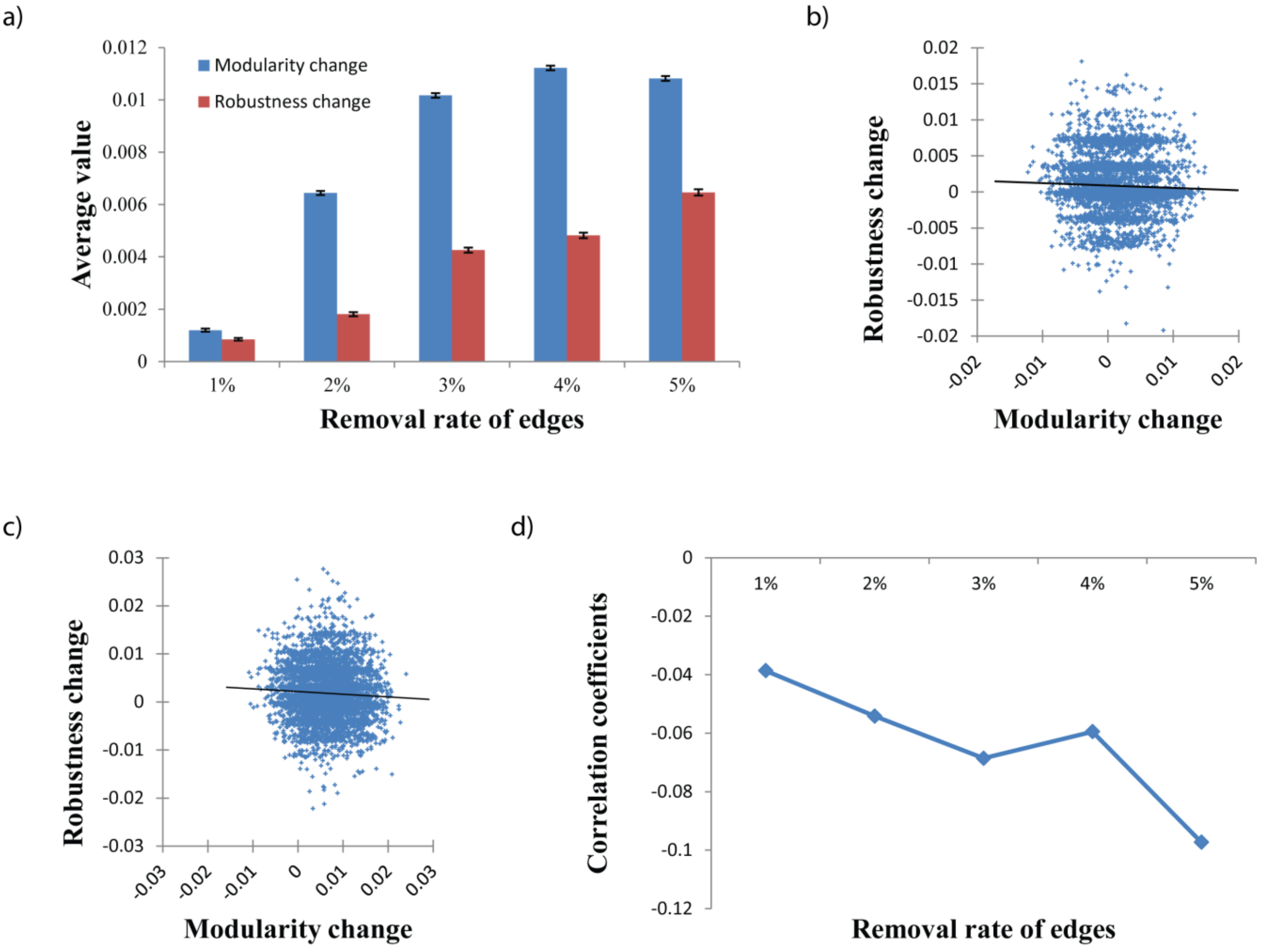

Fig. S2. Analysis of the changes of the modularity and the robustness by edge-removal mutations in HIV-1 signaling network. The removal rate of edges was varied from 1% to 5% (More specifically, the numbers of removed edges were 3, 7, 11, 14, and 18, respectively, among a total of 368 edges). For each removal rate, 5,000 trials of edge-removal were examined. **(a)** Results of average changes of the modularity and the robustness against the removal rate of edges. Y-axis value and error bar represent the average and the standard deviation divided by the square root of the sample size (5000), respectively. Both average values were significantly larger than zero (All P-values <0.0001 using one-sample t-test). The one-sample t-test was valid because the average values were normally distributed (see Additional file 1: Fig. S5) and there were very few outliers (see Additional file 1: Fig. S8). **(b)-(c)** Relationship between the changes of the modularity and the robustness in the case that the removal rate is 1% and 2%, respectively. A significant negative relationship was observed (Correlation coefficients were -0.03867 and -0.05417, respectively with all P-values <0.0001). This relationship was consistently observed for larger removal rates (Correlation coefficients when the edge-removal rate is 3%, 4%, and 5% were -0.06862 and, -0.05948, and -0.09733, respectively, with all P-values <0.0001). **(d)** A trend of correlation coefficients between the changes of the modularity and the robustness against the removal rate of edges.

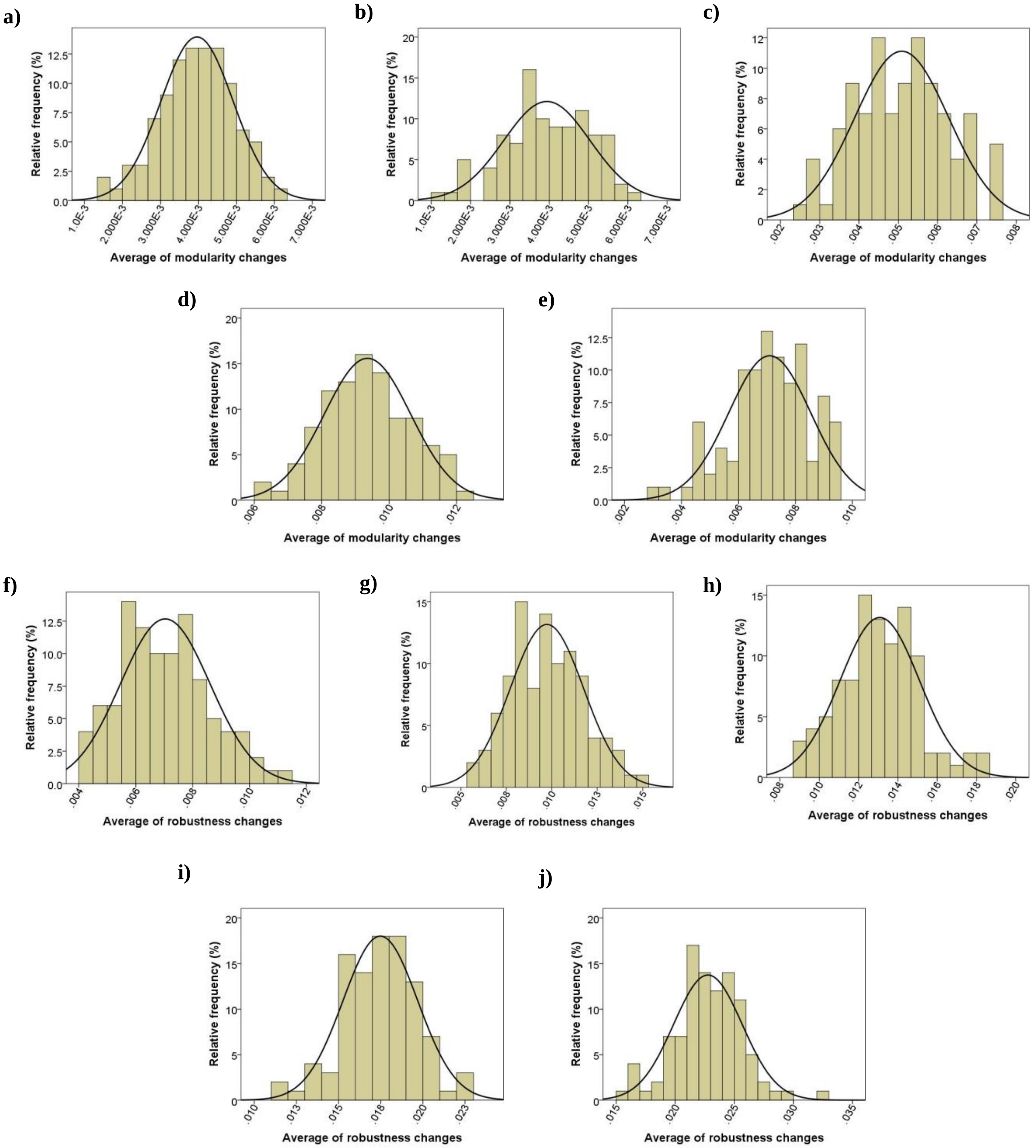

Fig. S3. Analysis of normal distributions of averages of modularity changes and robustness changes in T-LGL network. **(a)-(e)** Results of the average of the modularity change with removal rates of 1% to 5%, respectively. **(f)-(j)** Results of the average of the robustness change with removal rates of 1% to 5%, respectively. In each subfigure, the average of the modularity or the robustness changes over 50 trials is computed, and this process was repeated 100 times to examine the distribution of the average variable. Kolmogorov-Smirnov test was run and all average values were normally distributed (All P-values > 0.10).

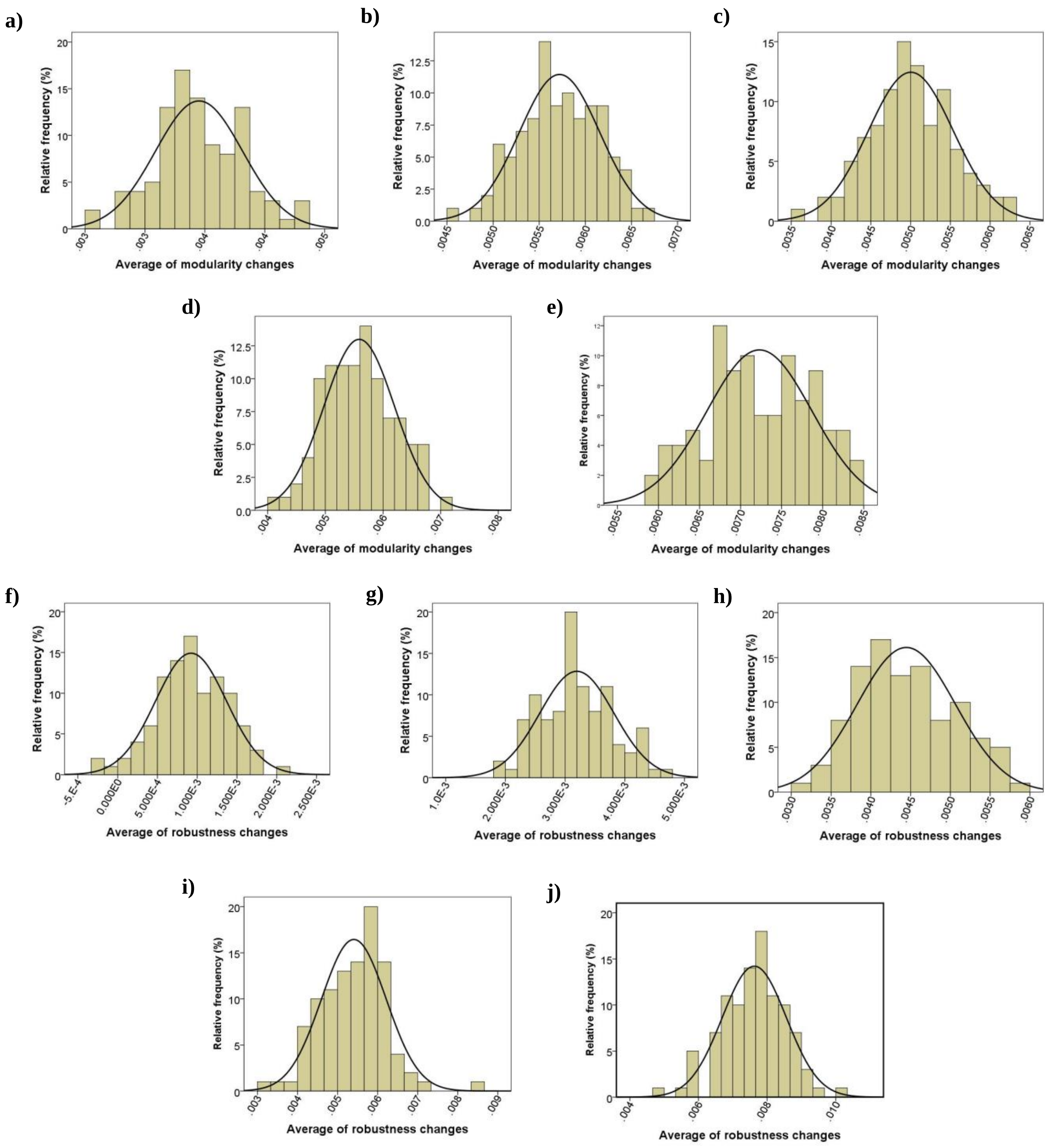

Fig. S4. Analysis of normal distributions of averages of modularity changes and robustness changes in STF network. **(a)-(e)** Results of the average of the modularity change with removal rates of 1% to 5%, respectively. **(f)-(j)** Results of the average of the robustness change with removal rates of 1% to 5%, respectively. In each subfigure, the average of the modularity or the robustness changes over 50 trials is computed, and this process was repeated 100 times to examine the distribution of the average variable. Kolmogorov-Smirnov test was run and all average values were normally distributed (All P-values > 0.10 except that P-value = 0.069 in (e)) .

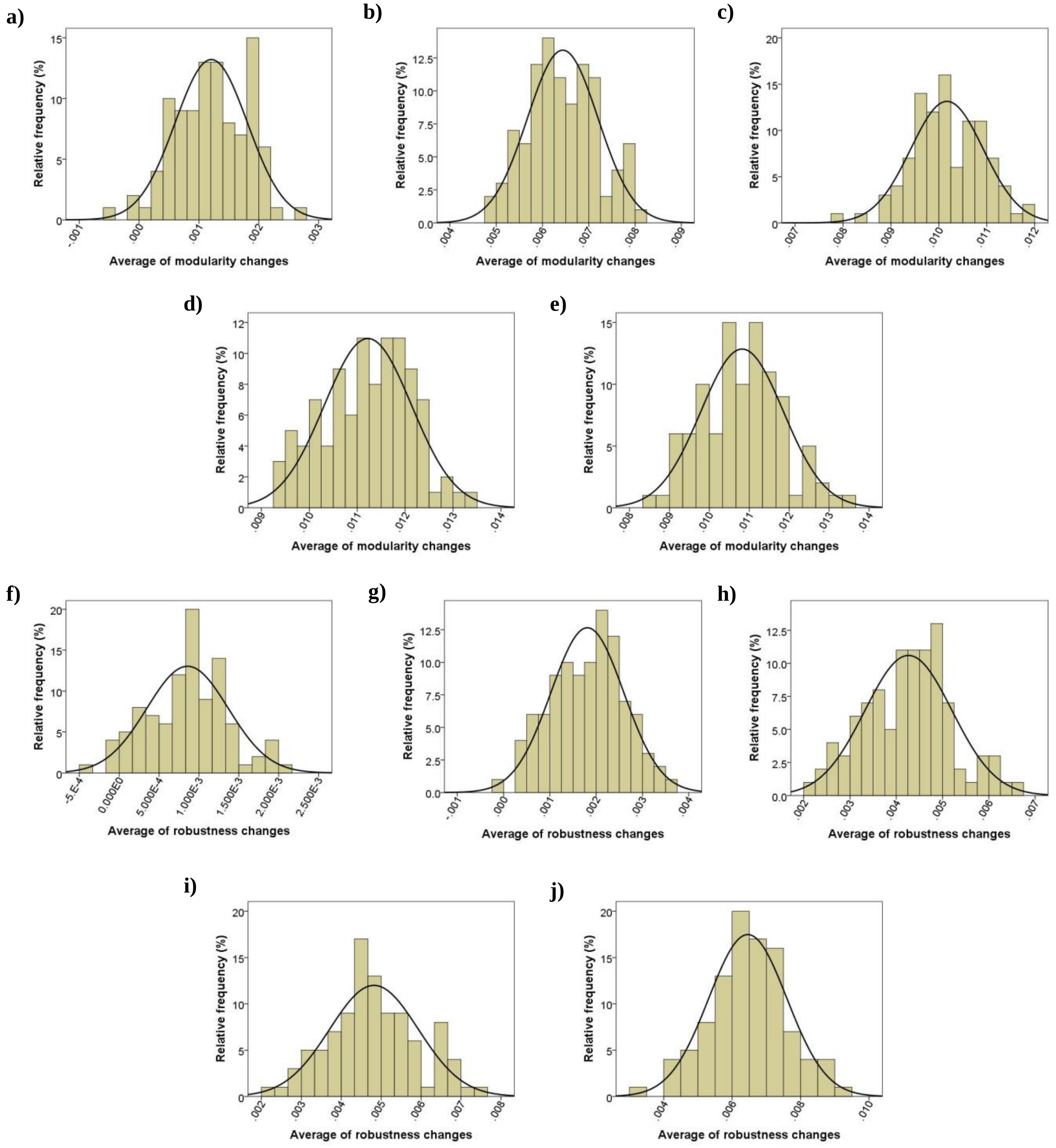

Fig. S5. Analysis of normal distributions of averages of modularity changes and robustness changes in HIV-1 network. **(a)-(e)** Results of the average of the modularity change with removal rates of 1% to 5%, respectively. **(f)-(j)** Results of the average of the robustness change with removal rates of 1% to 5%, respectively. In each subfigure, the average of the modularity or the robustness changes over 50 trials is computed, and this process was repeated 100 times to examine the distribution of the average variable. Kolmogorov-Smirnov test was run and all average values were normally distributed (All P-values > 0.10 except that P-value = 0.053 in (d)).

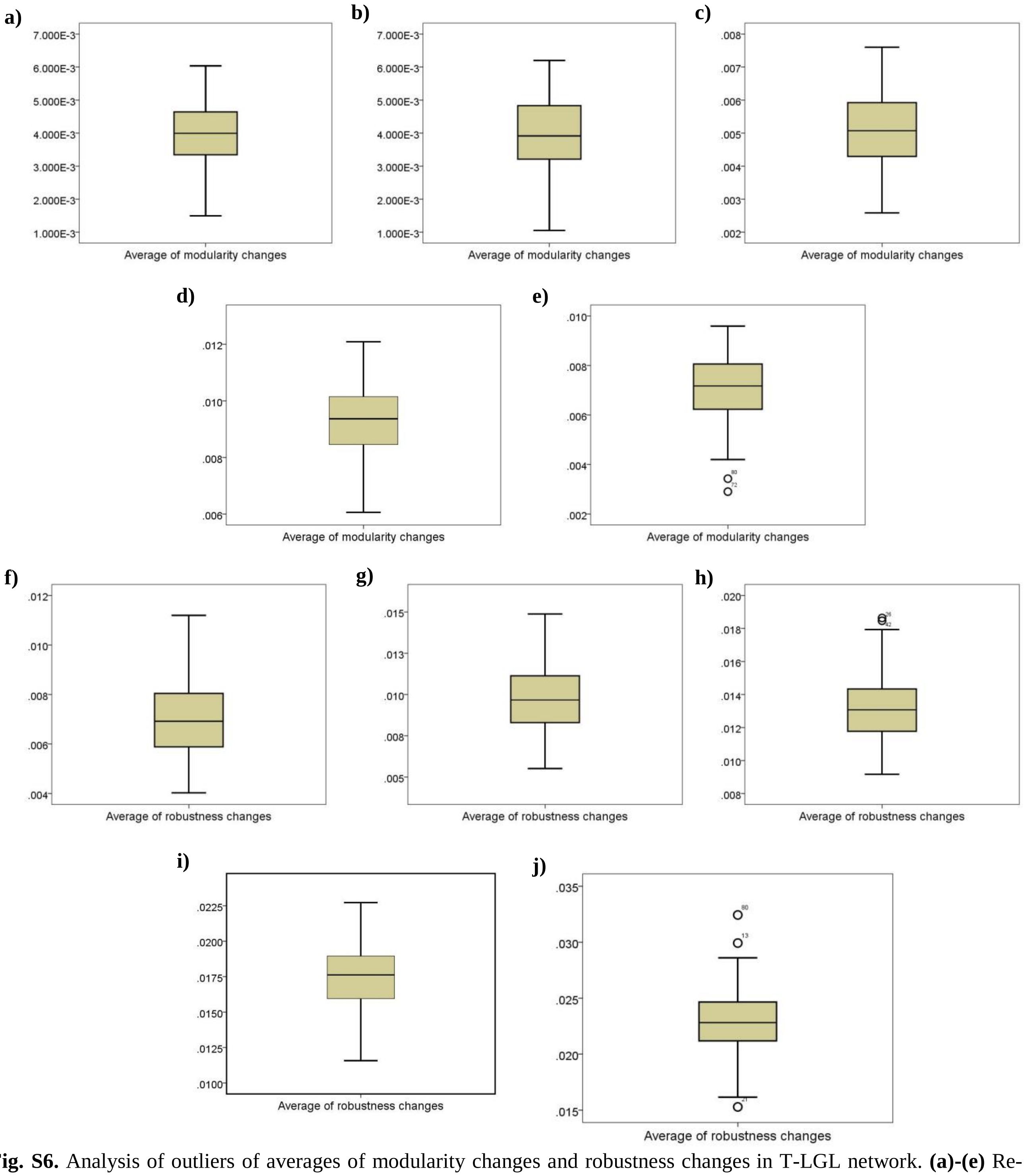

Fig. S6. Analysis of outliers of averages of modularity changes and robustness changes in T-LGL network. **(a)-(e)** Results of the average of the modularity change with removal rates of 1% to 5%, respectively. **(f)-(j)** Results of the average of the robustness change with removal rates of 1% to 5%, respectively. In each subfigure, the average of the modularity or the robustness changes over 50 trials is computed, and this process was repeated 100 times to examine the distribution of the average variable. We examined outliers by a boxplot inspection and found no significant outliers in all subfigures except for (e), (h) and (j).

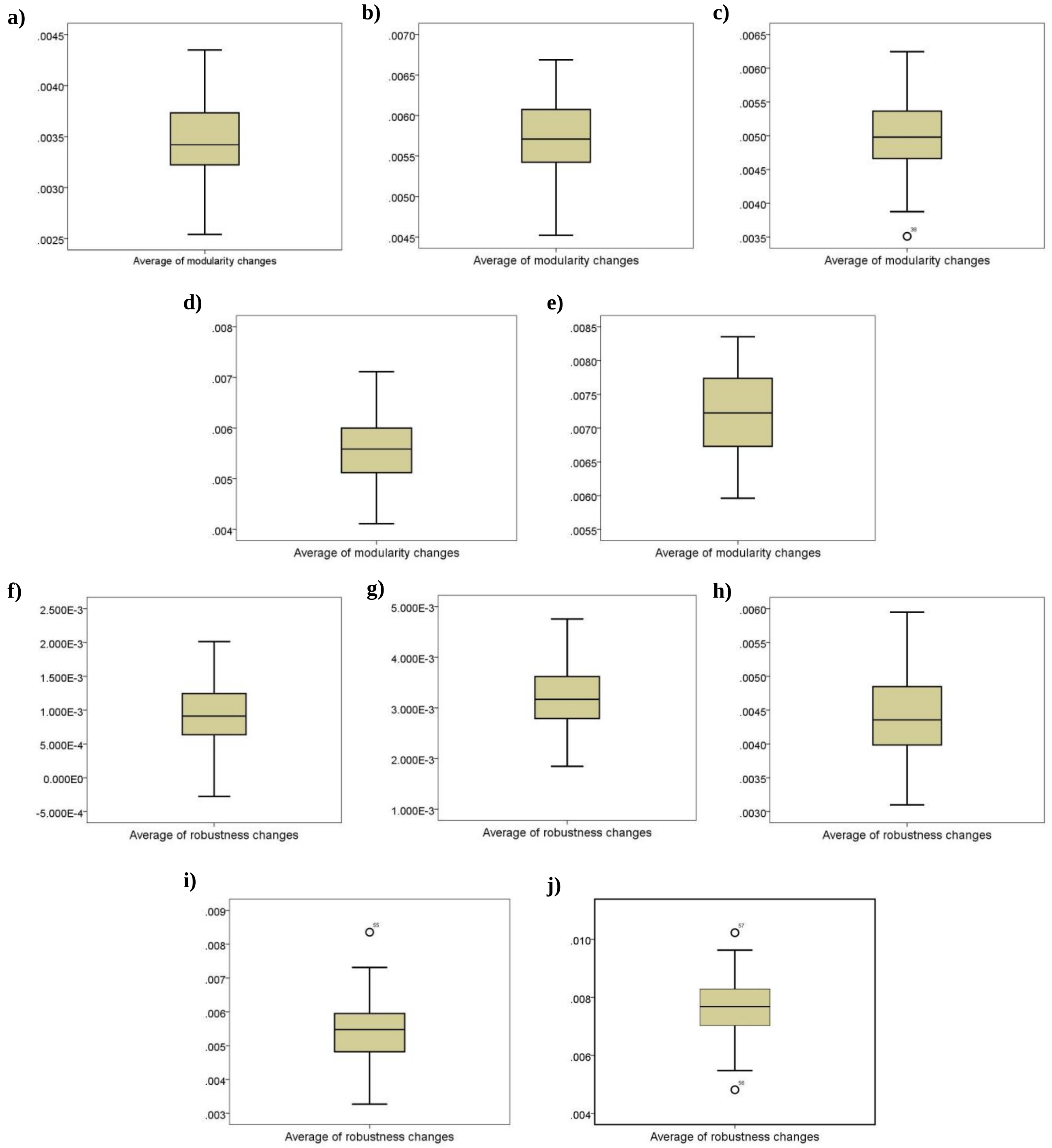

Fig. S7. Analysis of outliers of averages of modularity changes and robustness changes in STF network. **(a)-(e)** Results of the average of the modularity change with removal rates of 1% to 5%, respectively. **(f)-(j)** Results of the average of the robustness change with removal rates of 1% to 5%, respectively. In each subfigure, the average of the modularity or the robustness changes over 50 trials is computed, and this process was repeated 100 times to examine the distribution of the average variable. We examined outliers by a boxplot inspection and found no significant outliers in all subfigures except for (c), (i) and (j).

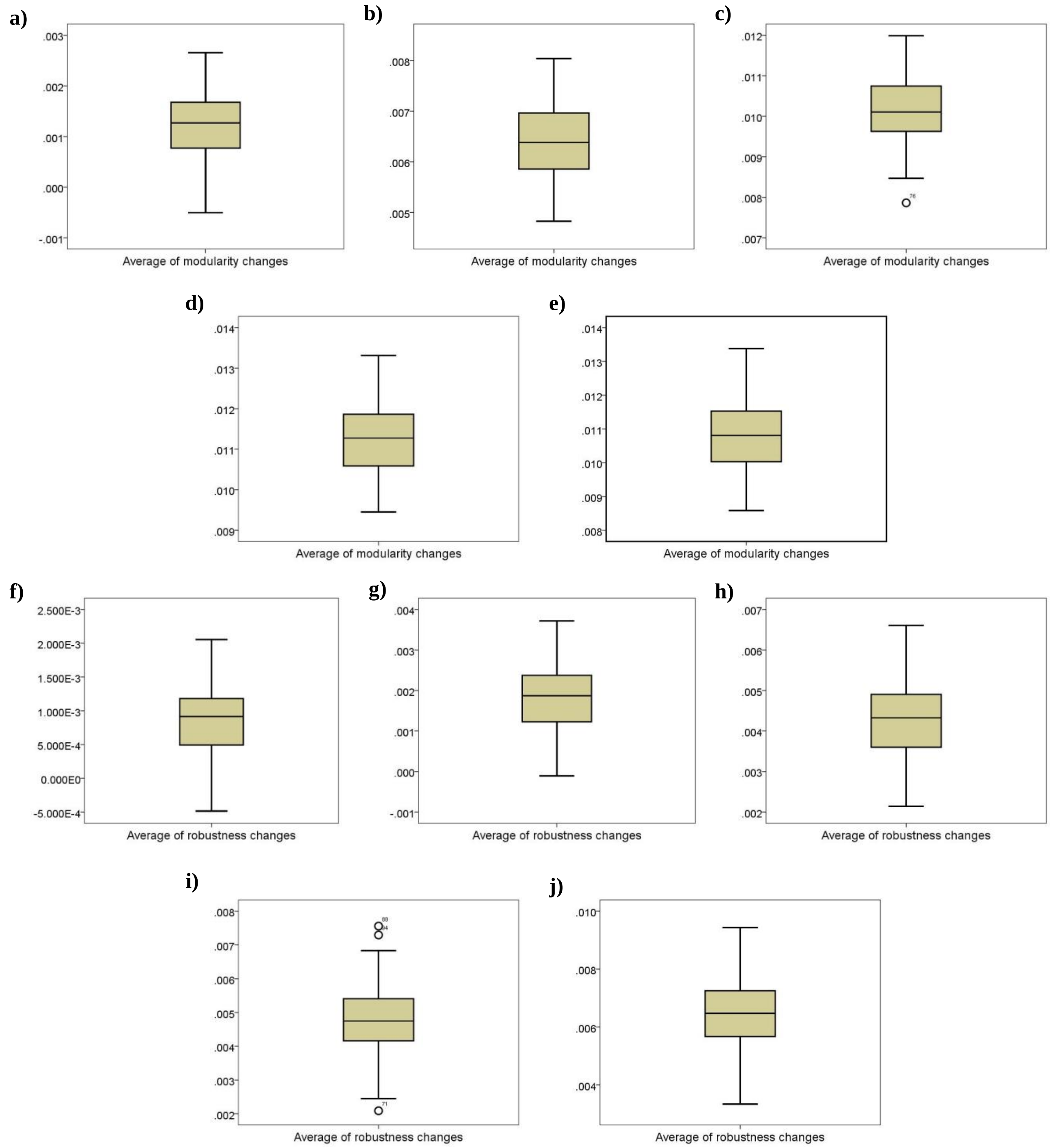

Fig. S8. Analysis of outliers of averages of modularity changes and robustness changes in HIV-1 network. **(a)-(e)** Results of the average of the modularity change with removal rates of 1% to 5%, respectively. **(f)-(j)** Results of the average of the robustness change with removal rates of 1% to 5%, respectively. In each subfigure, the average of the modularity or the robustness changes over 50 trials is computed, and this process was repeated 100 times to examine the distribution of the average variable. We examined outliers by a boxplot inspection and found no significant outliers in all subfigures except for (c) and (i).

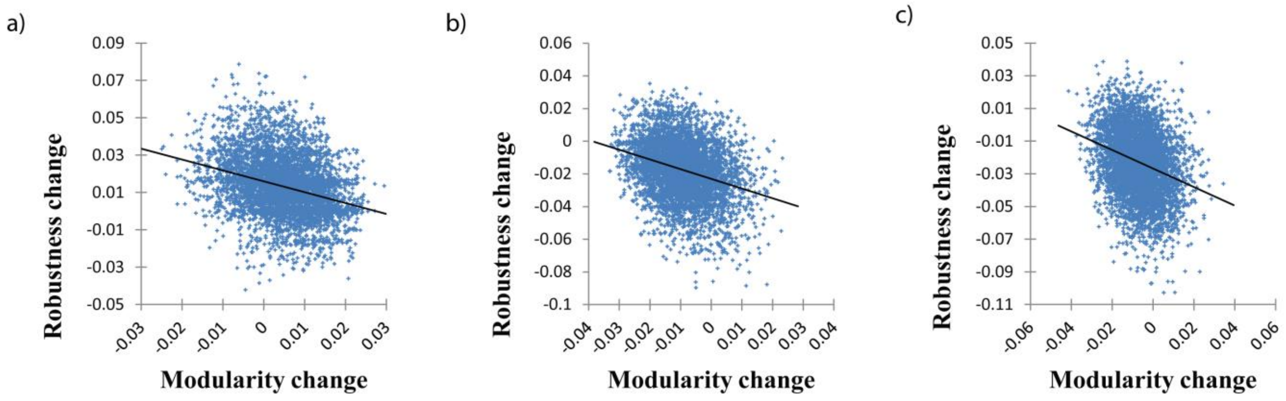

Fig. S9. Relationship between the changes of the modularity and the robustness in T-LGL signaling network. **(a)-(c)** Results in the cases that the edge-removal rate is 3%, 4%, and 5%, respectively. Each plot is a result of 5,000 trials. Correlation coefficients of (a)-(c) were -0.30652, -0.30684, and -0.28626, respectively (All P-values <0.0001).

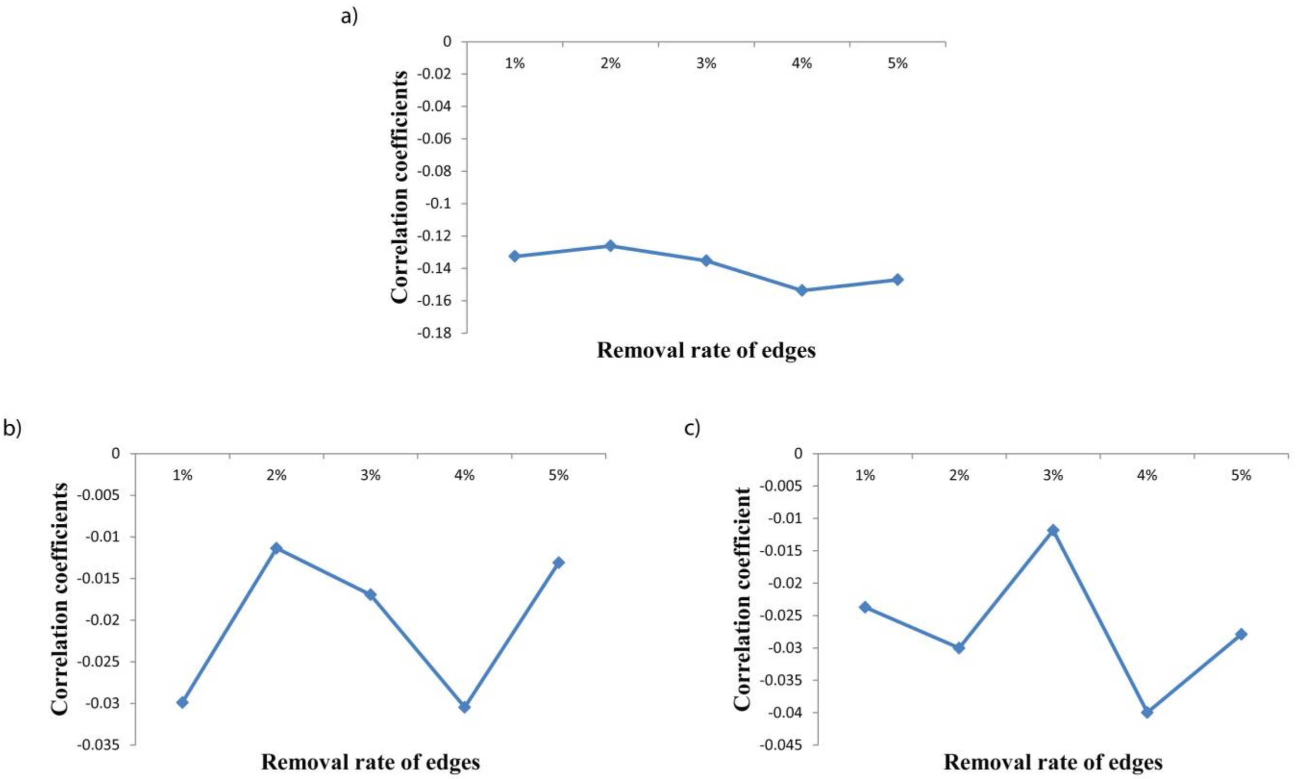

Fig. S10. Relationship between the changes of the modularity and the robustness in random networks. **(a)-(c)** Results of the random networks shuffled from T-LGL, STF, and HIV-1 signaling networks, respectively. In each subfigure, a set of 100 random networks were generated and 500 trials of edge-removals were tested for each network (Hence, each correlation coefficient was obtained over a total of 50,000 samples). We analyzed the relationship by varying the edge-removal rate from 1% to 5%. All cases showed significantly negative relationship (All P-values <0.0001).

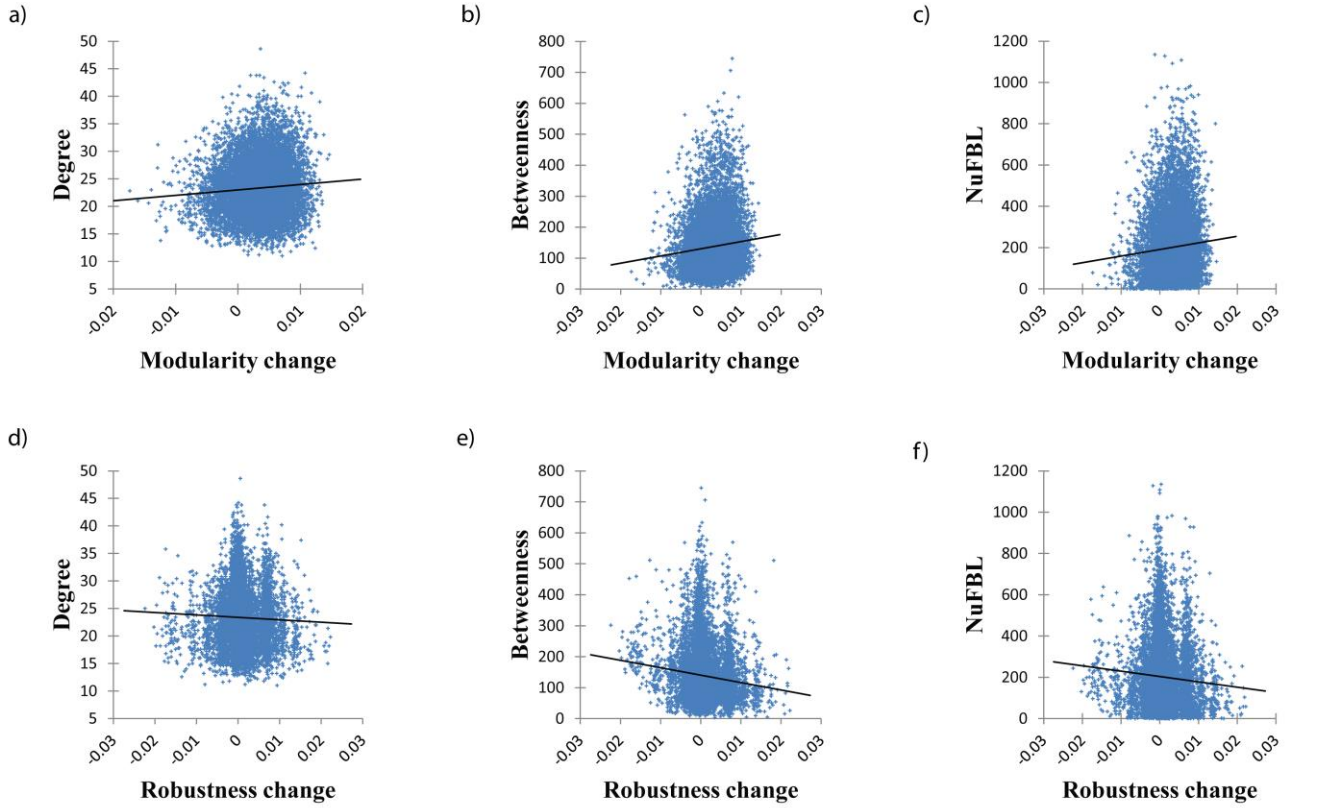

Fig. S11. Relationship of each of the changes of the modularity and the robustness with the structural properties in STF signaling network. The removal rate was set 1%, and a total of 5,000 trials of removals were examined. **(a)-(c)** Relations of the change of modularity with edge-based degree, EBEW, and NuFBL, respectively. The change of modularity was significantly positively correlated with all structural properties (Correlation coefficients were 0.07621, 0.10084, and 0.07762, respectively, with all P-values<0.0001). **(d)-(f)** Relations of the change of robustness with edge-based degree, EBEW, and NuFBL, respectively. The change of robustness was significantly negatively correlated with all structural properties (Correlation coefficients were -0.03749, -0.11430, and -0.06860, respectively, with all P-values<0.0001).

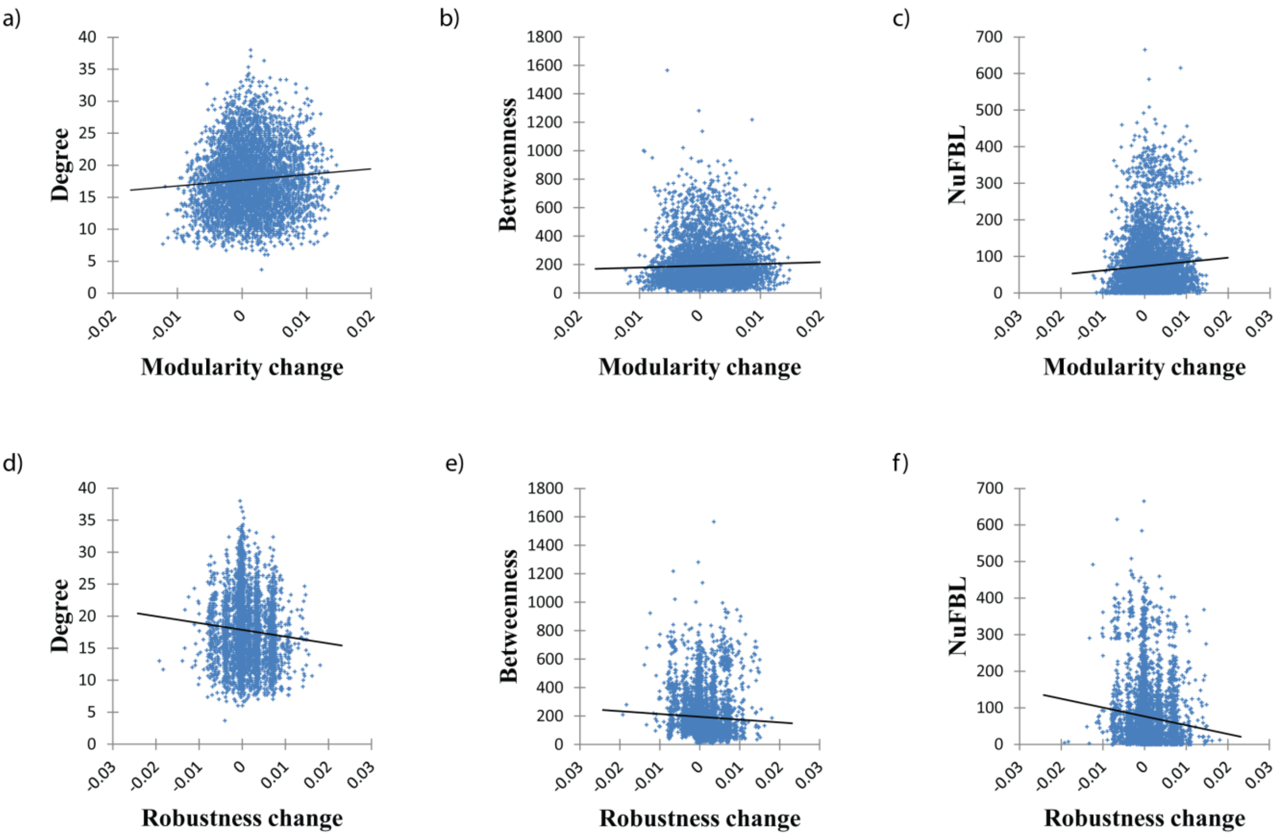

Fig. S12. Relationship of each of the changes of the modularity and the robustness with the structural properties in HIV-1 signaling network. The removal rate was set 1%, and a total of 5,000 trials of removals were examined. **(a)-(c)** Relations of the change of modularity with edge-based degree, EBEW, and NuFBL, respectively. The change of modularity was significantly positively correlated with all structural properties (Correlation coefficients were 0.07549, 0.03526, and 0.05970, respectively, with all P-values<0.0001). **(d)-(f)** Relations of the change of robustness with edge-based degree, EBEW, and NuFBL, respectively. The change of robustness was significantly negatively correlated with all structural properties (Correlation coefficients were -0.07664, -0.04671, and -0.10621, respectively, with all P-values<0.0001*).*

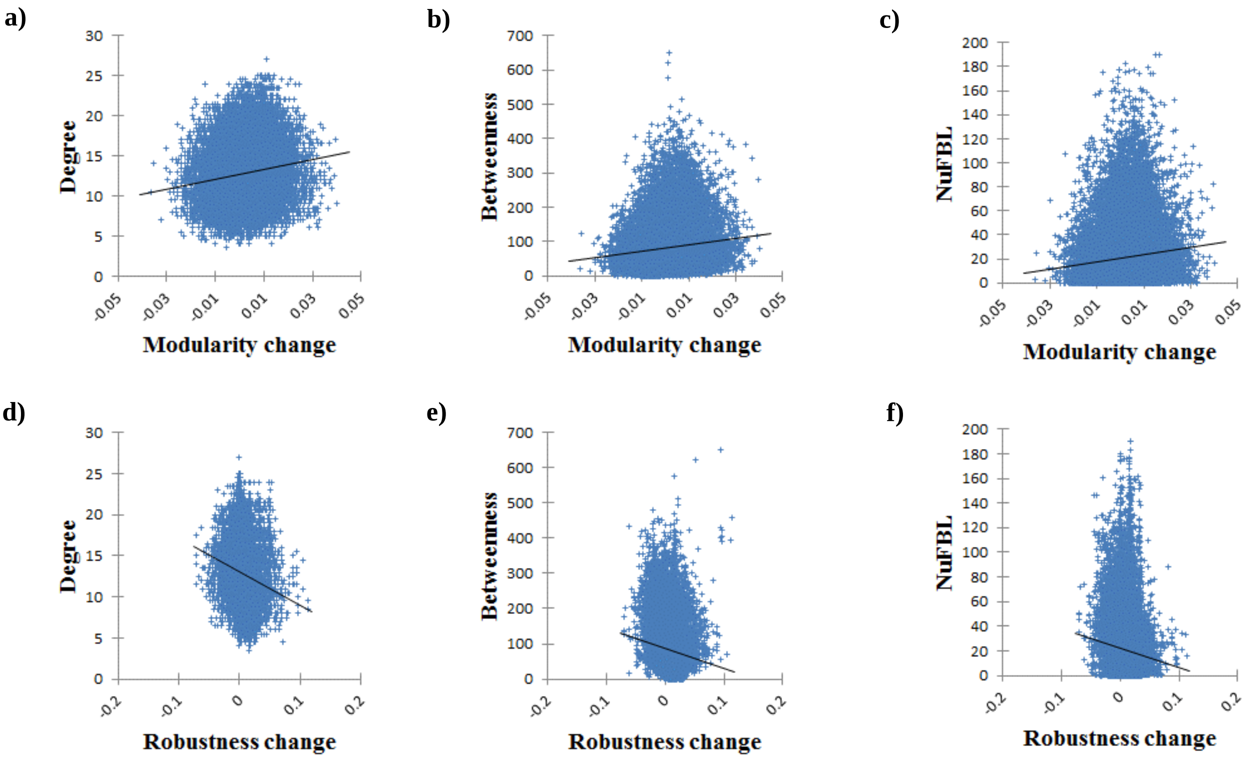

Fig. S13. Relationship of each of the changes of the modularity and the robustness with the structural properties in random networks shuffled from T-LGL network. In each subfigure, a set of 100 random networks were generated and 500 trials of edge-removal were tested for each network (Hence, each correlation coefficient was obtained over a total of 50,000 samples). The removal rate was set 1%. **(a)-(c)** Relations of the change of modularity with edge-based degree, EBEW, and NuFBL, respectively. The change of modularity was significantly positively correlated with all structural properties (Correlation coefficients were 0.14388, 0.11878, and 0.10775, respectively, with all P-values<0.0001). **(d)-(f)** Relations of the change of robustness with edge-based degree, EBEW, and NuFBL, respectively. The change of robustness was significantly negatively correlated with all structural properties (Correlation coefficients were -0.14851, -0.11281, and -0.08690, respectively, with all P-values<0.0001).

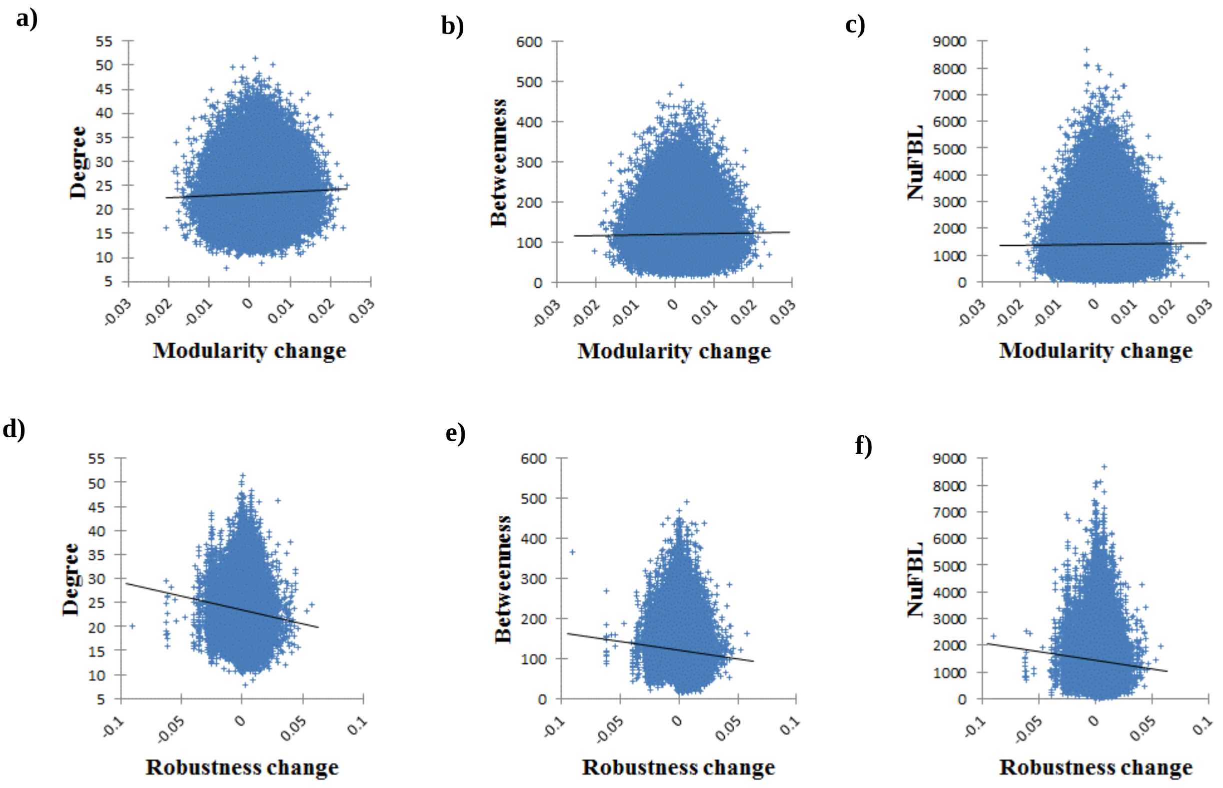

Fig. S14. Relationship of each of the changes of the modularity and the robustness with the structural properties in random networks shuffled from STF network. In each subfigure, a set of 100 random networks were generated and 500 trials of edge-removal were tested for each network (Hence, each correlation coefficient was obtained over a total of 50,000 samples). The removal rate was set 1%. **(a)-(c)** Relations of the change of modularity with edge-based degree, EBEW, and NuFBL, respectively. The change of modularity was significantly positively correlated with all structural properties (Correlation coefficients were 0.03228, 0.01516, and 0.01062, respectively, with all P-values<0.0001). **(d)-(f)** Relations of the change of robustness with edge-based degree, EBEW, and NuFBL, respectively. The change of robustness was significantly negatively correlated with all structural properties (Correlation coefficients were -0.07121, -0.05301, and -0.04716, respectively, with all P-values<0.0001).

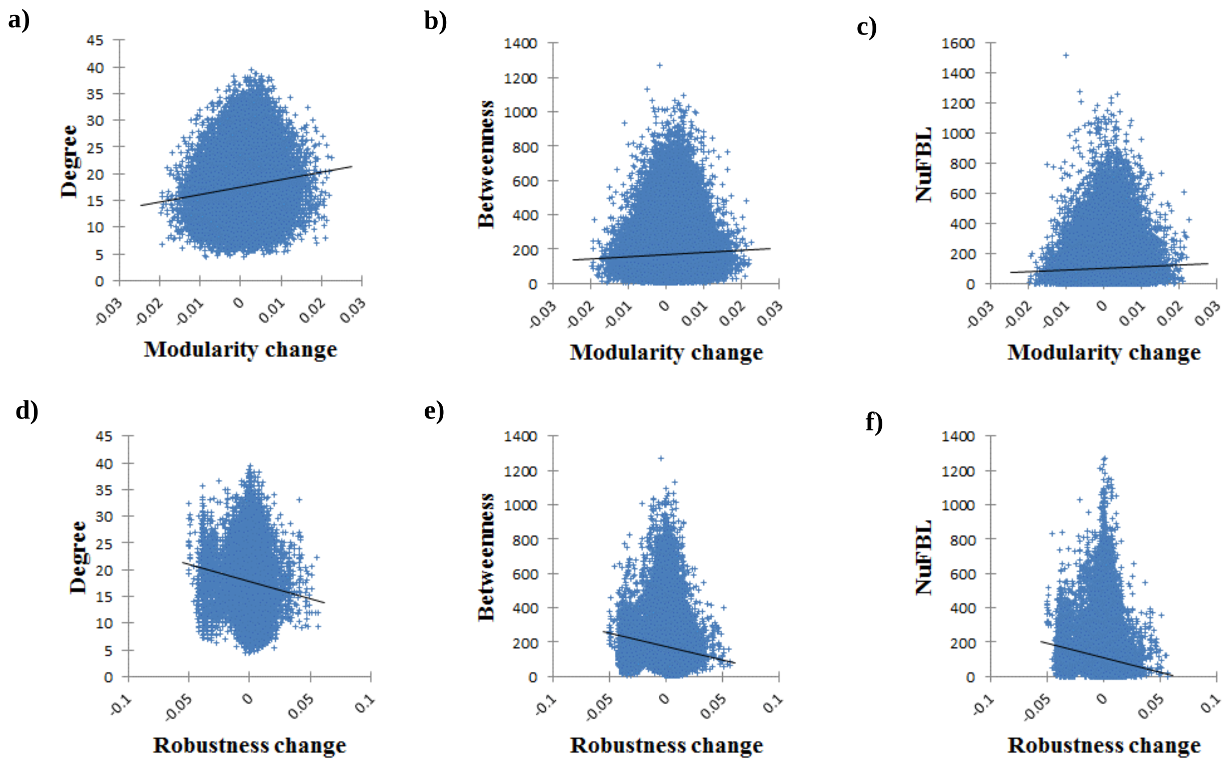

Fig. S15. Relationship of each of the changes of the modularity and the robustness with the structural properties in random networks shuffled from HIV-1 network. In each subfigure, a set of 100 random networks were generated and 500 trials of edge-removal were tested for each network (Hence, each correlation coefficient was obtained over a total of 50,000 samples). The removal rate was set 1%. **(a)-(c)** Relations of the change of modularity with edge-based degree, EBEW, and NuFBL, respectively. The change of modularity was significantly positively correlated with all structural properties (Correlation coefficients were 0.11393, 0.04112, and 0.04064, respectively, with all P-values<0.0001). **(d)-(f)** Relations of the change of robustness with edge-based degree, EBEW, and NuFBL, respectively. The change of robustness was significantly negatively correlated with all structural properties (Correlation coefficients were -0.08353, -0.08649, and -0.09906, respectively, with all P-values<0.0001).

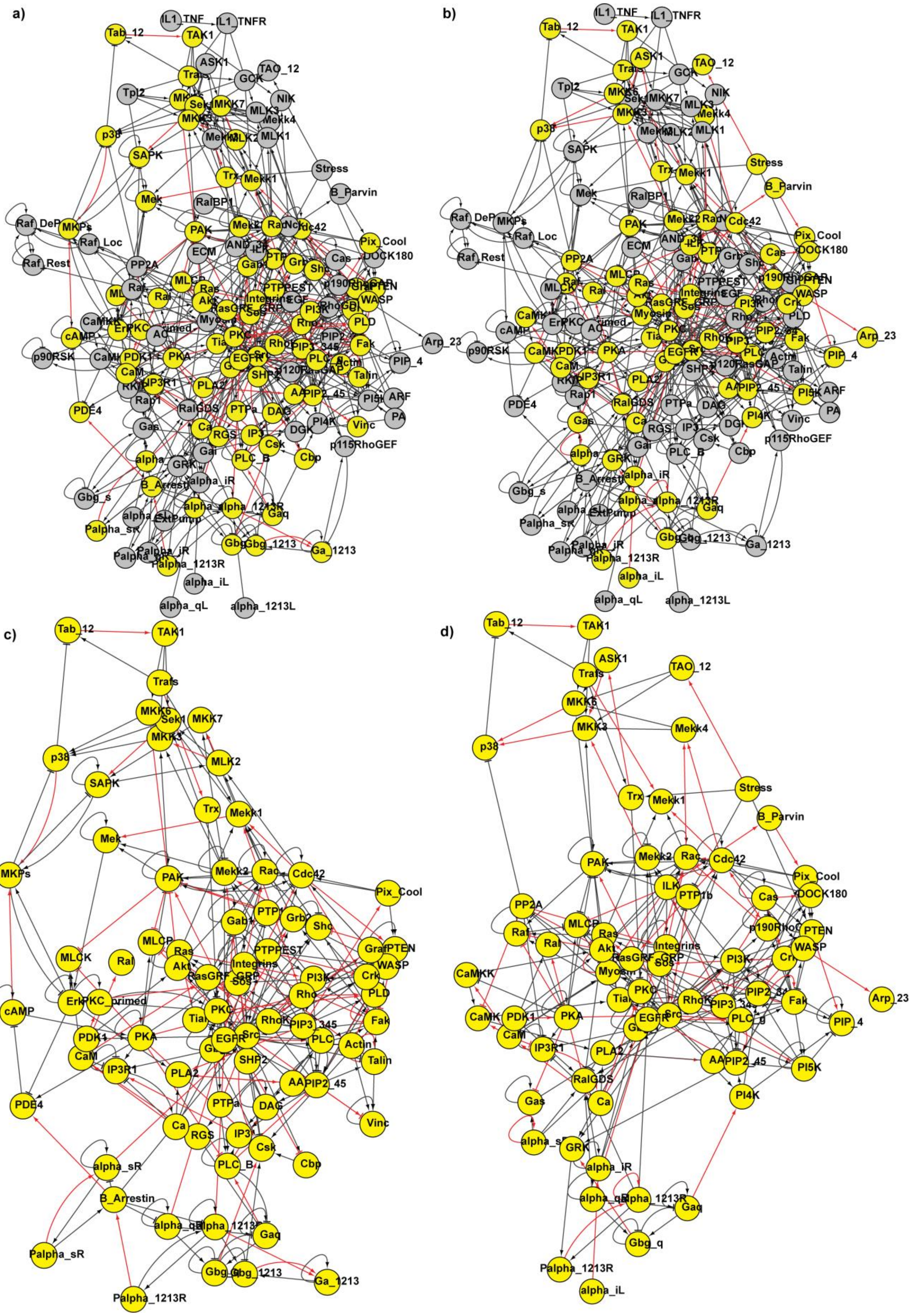

Fig. S16. Topological distributions of High-MI/High-RD edges and their incident nodes in STF signaling network. **(a)-(b)** Distributions of High-MI and High-RD edges, respectively, and their incident nodes. **(c)-(d)** Subgraphs with respect to High-MI-incident and High-RD-incident nodes, respectively. Red link and yellow node represent High-MI edge and High-MI-incident node, respectively, in both (a) and (c), whereas they represent High-RD edge and High-RD-incident node, respectively, in both (b) and (d).

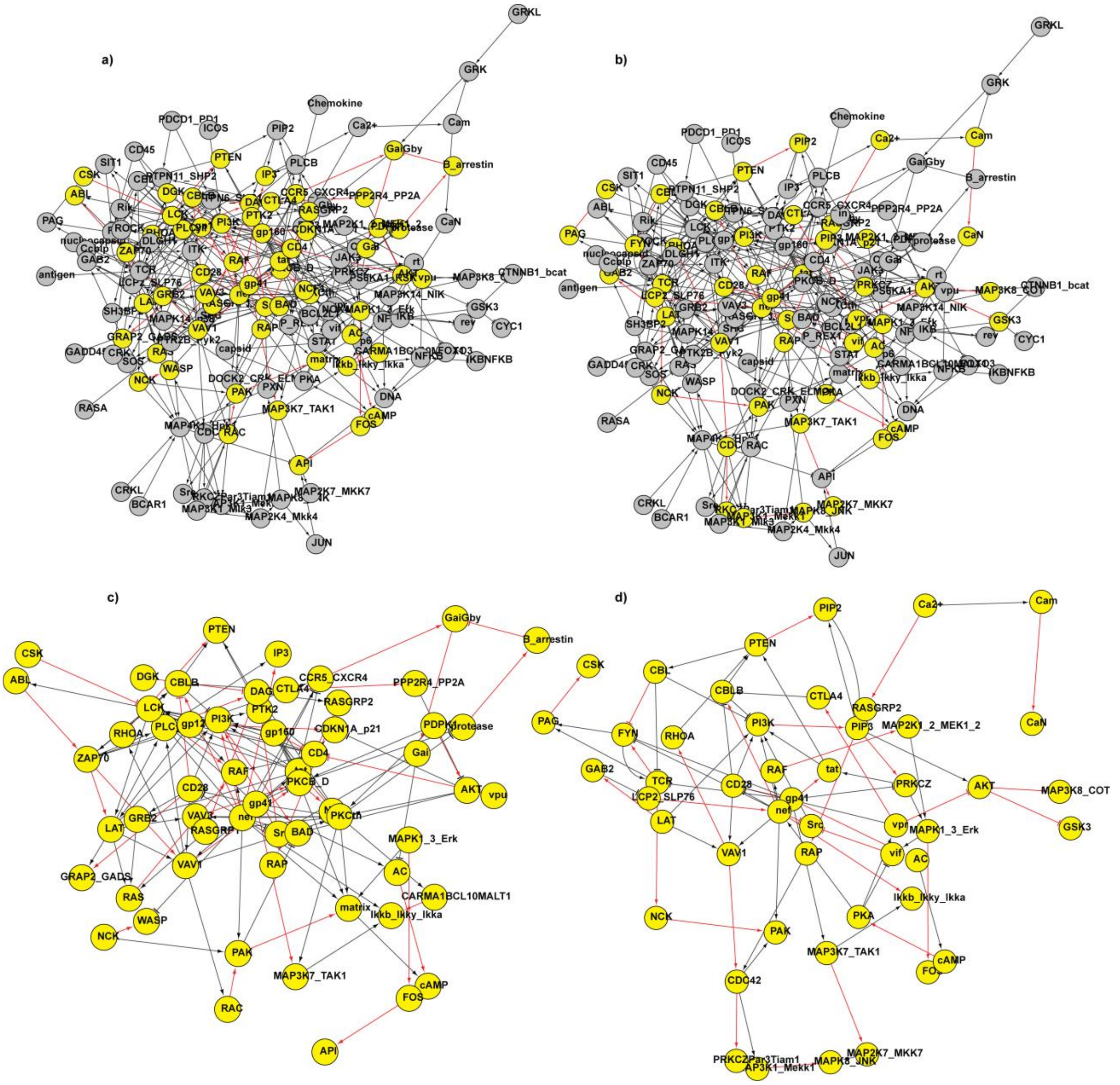

Fig. S17. Topological distributions of High-MI/High-RD edges and their incident nodes in HIV-1 signaling network. **(a)-(b)** Distributions of High-MI and High-RD edges, respectively, and their incident nodes. **(c)-(d)** Subgraphs with respect to High-MI-incident and High-RD-incident nodes, respectively. Red link and yellow node represent High-MI edge and High-MI-incident node, respectively, in both (a) and (c), whereas they represent High-RD edge and High-RD-incident node, respectively, in both (b) and (d).

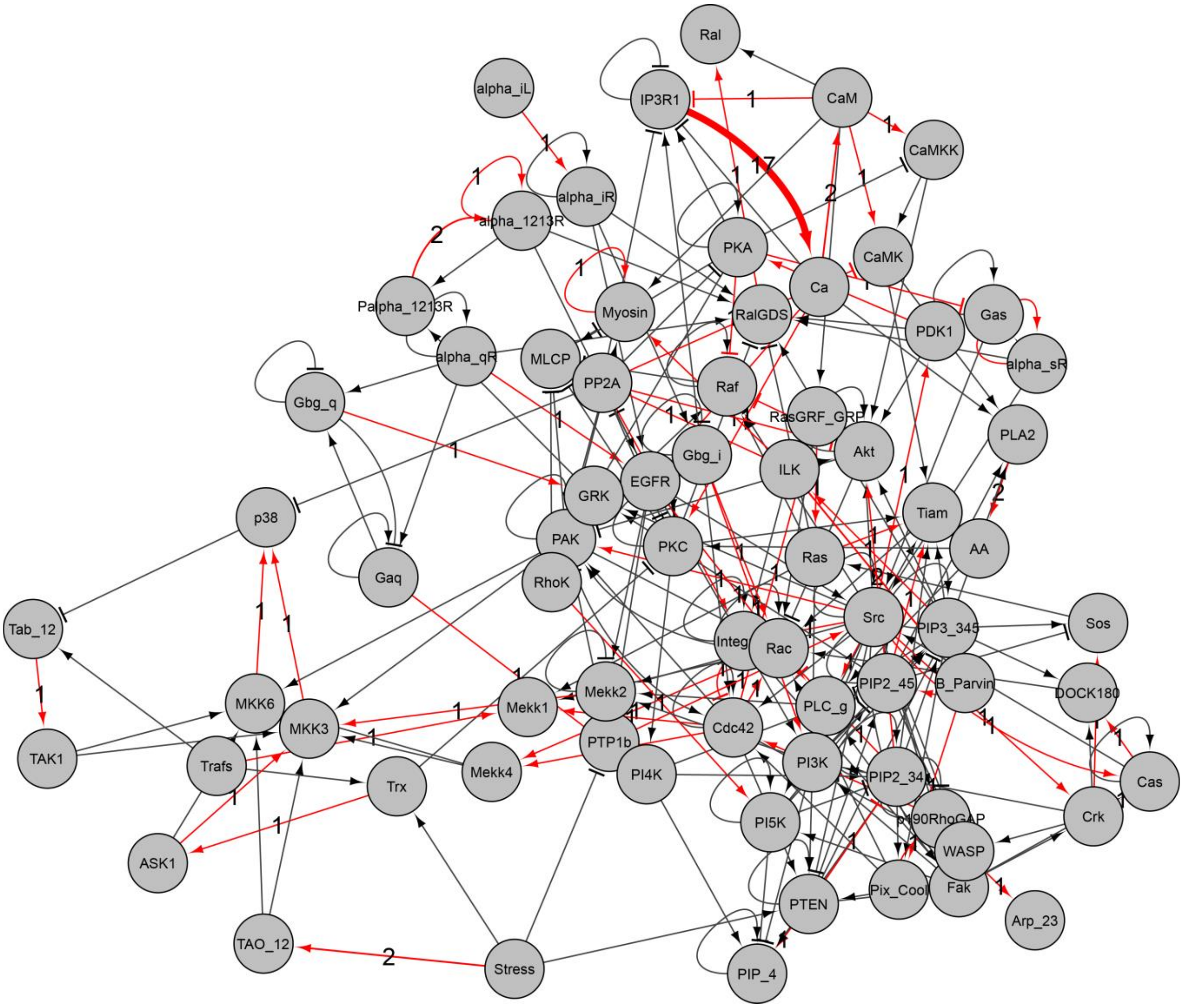

Fig. S18. Edge-removal analysis for edgetic drug discovery in STF signaling network. The arrows and bar-headed lines represent positive and negative interactions, respectively. Line thickness is proportional to the inclusion frequency of the interaction in top-K edge sets ranked in a decreasing order of the robustness change among 5000 trials of edge-removal mutations with 1% removal rate. The interaction ($IP3R1 \rightarrow Ca$) was observed 17 times in top-K edge sets (K was chosen to 18).

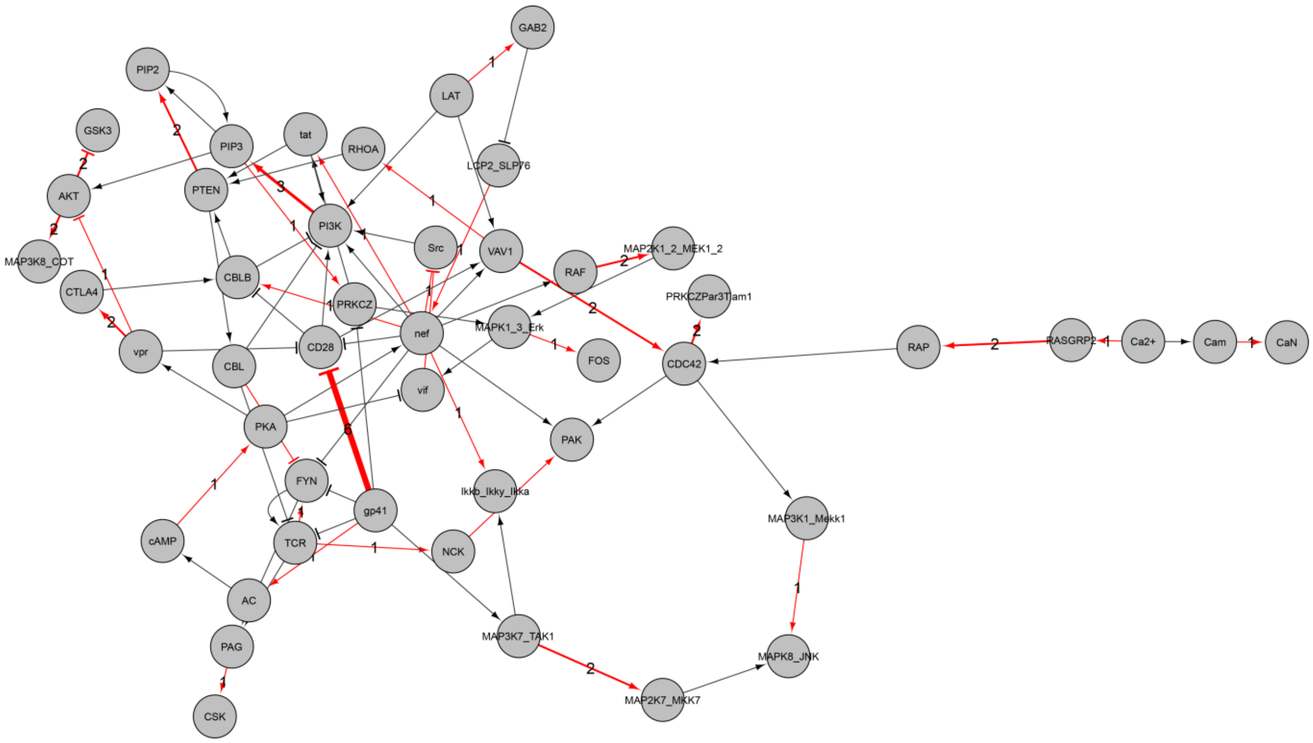

Fig. S19. Edge-removal analysis for edgetic drug discovery in HIV-1 signaling network. The arrows and bar-headed lines represent positive and negative interactions, respectively. Line thickness is proportional to the inclusion frequency of the interaction in top-*K* edge sets ranked in a decreasing order of the robustness change among 5000 trials of edge-removal mutations with 1% removal rate. The interactions ($gp41 \rightarrow CD28$) and ($PI3K \rightarrow PIP3$) were observed 6 and 3 times, respectively, in top-*K* edge sets (*K* was chosen to 16).

Table. S1. GO analysis results between High-MI-incident/High-RD-incident group and the rest of genes in STF network. All P-values were calculated by using Bonferroni test.

Type of GO analysis	GO term	High-MI-incident (%)	The rest of genes (%)	P-value
Modularity change	Protein kinase activator activity	75.00	25.00	360.0E-6
	Negative regulation of cysteine-type endopeptidase activity	66.84	40.11	1.1E-6
	Response to mechanical stimulus	74.13	37.07	93.0E-9
	Activation of immune response	75.09	27.66	120.0E-18
	Protein phosphatase binding	84.82	21.21	7.1E-9
	Mitogen-activated protein kinase kinase kinase binding	75.00	25.00	1.9E-6
	Growth factor receptor binding	76.73	30.69	50.0E-6
	Insulin receptor binding	89.82	17.96	450.0E-9
	GO term	High-RD-incident (%)	The rest of genes (%)	P-value
Robustness change	Rho guanyl-nucleotide exchange factor activity	75.00	25.00	760.0E-6
	Protein serine/threonine/tyrosine kinase activity	62.50	37.50	44.0E-12
	Cellular response to carbohydrate stimulus	66.67	33.33	47.0E-6
	Response to epidermal growth factor	60.00	40.00	1.4E-6
	Antigen receptor-mediated signaling pathway	72.73	27.27	42.0E-9
	Lipopolysaccharide-mediated signaling pathway	75.00	25.00	190.0E-6
	Immune response-activating cell surface receptor signaling pathway	71.70	31.37	55.0E-18
	Neurotrophin TRK receptor signaling pathway	64.60	43.07	8.5E-6

Table. S2. GO analysis results between High-MI-incident/High-RD-incident group and the rest of genes in HIV-1 network. All P-values were calculated by using Bonferroni test.

Type of GO analysis	GO term	High-MI-incident (%)	The rest of genes (%)	P-value
Modularity change	Negative regulation of kinase activity	61.54	38.46	720.0E-12
	Virus receptor activity	75.00	25.00	470.0E-6
	Regulation of defense response to virus	60.00	40.00	100.0E-6
	Positive regulation of immune response	64.34	39.06	560.0E-36
	Response to growth hormone	66.67	33.33	45.0E-9
	Phosphatase binding	63.64	36.36	510.0E-12
	Phosphotyrosine binding	60.00	40.00	5.5E-9
	Protein phosphatase binding	66.67	33.33	7.9E-9
	GO term	High-RD-incident (%)	The rest of genes (%)	P-value
Robustness change	Receptor signaling protein serine/threonine kinase activity	72.73	27.27	2.5E-12
	MAP kinase kinase kinase activity	75.00	25.00	7.0E-6
	Response to axon injury	75.00	25.00	550.0E-6
	Mast cell activation involved in immune response	61.39	46.04	200.0E-9
	Stimulatory C-type lectin receptor signaling pathway	60.00	40.00	180.0E-12
	Lipopolysaccharide-mediated signaling pathway	60.00	40.00	11.0E-6
	Negative regulation of ERBB signaling pathway	75.00	25.00	91.0E-6
	Negative regulation of epidermal growth factor receptor signaling pathway	75.00	25.00	83.0E-6

References

Kauffman, S., *et al.* (2003) Random Boolean network models and the yeast transcriptional network, *P Natl Acad Sci USA*, **100**, 14796-14799.

Kauffman, S., *et al.* (2004) Genetic networks with canalyzing Boolean rules are always stable, *P Natl Acad Sci USA*, **101**, 17102-17107.
